# Enhancement of the gut barrier integrity by a microbial metabolite through the Nrf2 pathway

**DOI:** 10.1038/s41467-018-07859-7

**Published:** 2019-01-09

**Authors:** Rajbir Singh, Sandeep Chandrashekharappa, Sobha R. Bodduluri, Becca V. Baby, Bindu Hegde, Niranjan G. Kotla, Ankita A. Hiwale, Taslimarif Saiyed, Paresh Patel, Matam Vijay-Kumar, Morgan G. I. Langille, Gavin M. Douglas, Xi Cheng, Eric C. Rouchka, Sabine J. Waigel, Gerald W. Dryden, Houda Alatassi, Huang-Ge Zhang, Bodduluri Haribabu, Praveen K. Vemula, Venkatakrishna R. Jala

**Affiliations:** 1 Department of Microbiology and Immunology, James Graham Brown Cancer Center, University of Louisville, Louisville, KY 40202 USA; 2 Institute for Stem Cell Biology and Regenerative Medicine (inStem), GKVK campus, Bangalore, Karnataka 560065 India; 3 Centre for Cellular and Molecular Platforms (C-CAMP), GKVK campus, Bangalore, Karnataka 560065 India; 4 Department of Physiology and Pharmacology, University of Toledo College of Medicine and Life Sciences, Toledo, OH 43614 USA; 5 Department of Pharmacology, Dalhousie University, Halifax, B3H 4R2 Nova Scotia Canada; 6 Computer Engineering and Computer Science, Kentucky Biomedical Research Infrastructure Network, University of Louisville, Louisville, KY 40202 USA; 7 Department of Medicine, University of Louisville, Louisville, KY 40202 USA; 8 Department of Pathology, University of Louisville, Louisville, KY 40202 USA

## Abstract

The importance of gut microbiota in human health and pathophysiology is undisputable. Despite the abundance of metagenomics data, the functional dynamics of gut microbiota in human health and disease remain elusive. Urolithin A (UroA), a major microbial metabolite derived from polyphenolics of berries and pomegranate fruits displays anti-inflammatory, anti-oxidative, and anti-ageing activities. Here, we show that UroA and its potent synthetic analogue (UAS03) significantly enhance gut barrier function and inhibit unwarranted inflammation. We demonstrate that UroA and UAS03 exert their barrier functions through activation of aryl hydrocarbon receptor (AhR)- nuclear factor erythroid 2–related factor 2 (Nrf2)-dependent pathways to upregulate epithelial tight junction proteins. Importantly, treatment with these compounds attenuated colitis in pre-clinical models by remedying barrier dysfunction in addition to anti-inflammatory activities. Cumulatively, the results highlight how microbial metabolites provide two-pronged beneficial activities at gut epithelium by enhancing barrier functions and reducing inflammation to protect from colonic diseases.

## Abstract (web summary)

Urolithins are microbial metabolites derived from food polyphenols. Here, Singh et al. show that urolithin A and a synthetic analogue enhance gut barrier function via Nrf2-dependent pathways and mitigate inflammation and colitis in mice, highlighting a potential application for inflammatory bowel diseases.

## Introduction

Inflammatory bowel diseases (IBD) consisting of Crohn’s and ulcerative colitis are resultant of dysregulation of the immune system leading to intestinal inflammation and microbial dysbiosis. Numerous studies in recent years highlighted the pivotal role of gut microbiota and their metabolites in host physiological processes including immune, metabolic, neurological, and nutritional homeostasis^1–4^. Thus, alterations in gut microbiota have been associated with adverse outcomes in cancer, IBD, neurological disorders, obesity, and diabetes^1,5–7^. Microbiota and their metabolites are in close proximity to the gut epithelium that constitutes a single cell-layer separating host components from the external environment. Gut barrier integrity is maintained by the tight junction proteins such as claudins (Cldn), Zona occludin -1 (ZO1), and occludin (Ocln) that are critical for epithelial cell barrier functions^8,9^. Previously, it has been reported that levels of tight junction proteins are significantly down regulated under IBD conditions leading to increased gut permeability to microbial ligands and noxious metabolites resulting in systemic inflammatory responses^8,10^. Despite the availability of large metagenomics data, the functional dynamics of microbiota and their metabolites in IBDs are unknown. Therefore, we tested the hypothesis that certain microbial metabolites will prevent gut permeability by enhancing barrier functions in addition to blocking inflammation. Treatment with such microbial metabolites will offer better therapeutic options for IBDs.

Consumption of diets containing berries and pomegranates have demonstrated significant beneficial effects on human health^11–14^. Especially, pomegranate extract or juice containing high levels of polyphenolic compounds such as ellagitannins (ETs) and ellagic acid (EA) have been suggested to prevent hypertension^15^ and protect against myocardial ischemia and reperfusion injury^16^. They have been recognized as potential non-toxic chemo-preventive compounds against chronic diseases such as cancer, diabetes, cardiovascular and neurodegenerative disorders^17^. It has been suggested that further downstream metabolites of EA known as ‘urolithins’ generated by gut microbiota render potential health benefits, in vivo^18,19^. Among urolithins, Urolithin A (UroA) displayed potent anti-inflammatory, anti-oxidative and anti-ageing properties compared to other metabolites^20–23^. Due to life style variations and antibiotic/drug usage, presence of bacteria that metabolize dietary EA to urolithins have been variable among human populations. Thus, not only the consumption of diets enriched in polyphenols is required but also the presence of microbes that convert them into beneficial metabolites is critical for manifestation of their health effects. At this time, the targets or pathways through which such microbial metabolites regulate physiological processes are largely unknown.

In this study, we examined the activities of UroA and a potent synthetic structural analogue UAS03 and identified that in addition to the anti-inflammatory activities, these compounds strongly enhanced gut barrier function. We demonstrate that both UroA and UAS03 enhance barrier function by inducing tight junction proteins through activating aryl hydrocarbon receptor (AhR)-nuclear factor erythroid 2–related factor 2 (Nrf2)-dependent pathways. Further, oral treatment with UroA/UAS03 significantly mitigated systemic inflammation and colitis suggesting potential therapeutic applications for the treatment of IBD.

## Results

### Synthesis and anti-inflammatory activities of UroA and UAS03

**Fig. 1:**
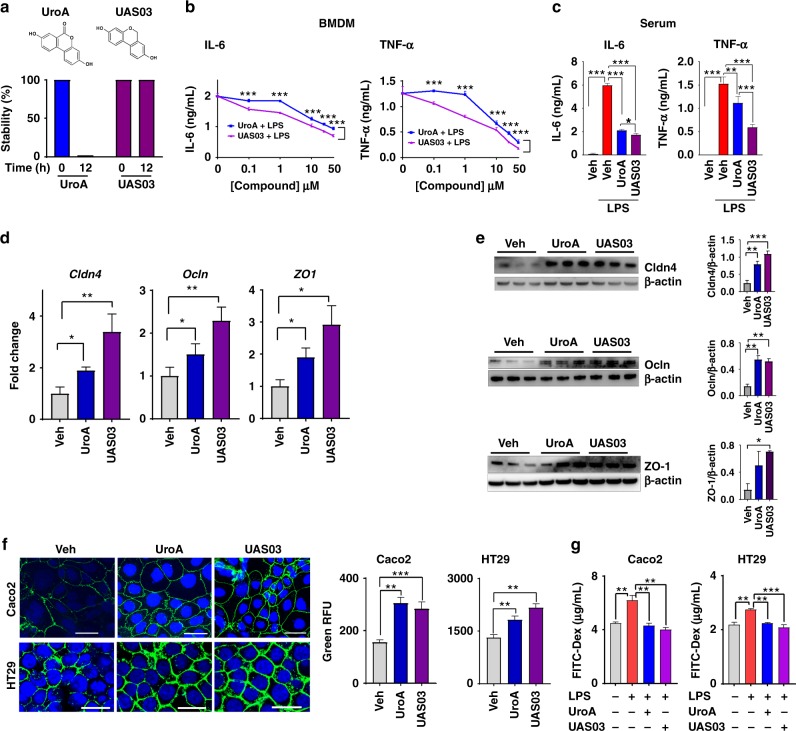
UAS03 is a potent anti-inflammatory structural analogue of UroA and induces tight junction proteins. **a** Chemical structures of UroA, UAS03. UroA/UAS03 stability was examined in the presence of gastric pH 2.0 and digestive enzymes. UroA and UAS03 (0.2 mg/ml) were incubated with digestive enzymes (esterases and proteases, 100 U/ml) for 12 h at 37 °C and compound levels were quantified. **b** BMDMs were stimulated with LPS (50 ng/ml) without or with UroA (blue line)/UAS03 (purple line) (0.1, 1, 10, 25, and 50 µM) for 6 h. IL-6 and TNF-α levels in supernatants were measured. **c** C57BL/6 mice (n = 3–4) were pretreated with UroA (20 mg/kg) and UAS03 (20 mg/kg). After 4 h, LPS (2 mg/kg) was injected intraperitoneally. Post 4 h of LPS administration, serum levels of IL-6 and TNF-α was measured. **d**–**f** HT29 or Caco2 cells were treated with vehicle (DMSO-0.01%) or UroA/UAS03 (50 μM) for 24 h. **d** The fold changes in mRNA levels of claudin 4 (*Cldn4*), occludin (*Ocln*), and Zona occludens 1 (*ZO1*) in HT29 cells were determined by RT PCR method. **e** UroA/UAS03 induced protein expression of Cldn4, Ocln, and ZO1 in HT29 cells were determined by immunoblots and quantified by Image J software. **f** Caco2 or HT29 cells were grown on coverslip bottom FluroDish and treated with Vehicle, UroA/UAS03 for 24 h. The cells were stained with anti-Cldn4 followed by secondary antibody tagged with Alexa-488. Nucleus was stained using DAPI. The confocal images were captured. The green intensity (*n* = 15–20 cell membrane regions) was measured. Scale bars for Caco2 and HT29 cells indicate 50 and 25 μm respectively. **g** Monolayer HT29 or Caco2 cells on transmembranes were treated with vehicle or UroA/UAS03 (50 μM) for 24 h followed by treatment with LPS (50 ng/ml) for 2 h. FITC-dextran was added to these cells (top of the membrane) and incubated for 2 h and FITC-dextran levels in bottom chamber well was measured. Results are representative of three independent experiments with triplicates for each concentration. *p < 0.05, **p < 0.01, ***p < 0.001, unpaired *t*-test between Veh, UroA, or UAS03. Error bars, ±SEM. Source Data are provided as a Source Data File

UroA (3,8-dihydroxy-6H-dibenzo[b,d]pyran-6-one) has a lactone (cyclic ester bond) that connects two mono-hydroxyl phenyl rings leading to a planar structure (Fig. 1a). Gastric pH or digestive enzymes can hydrolyze the lactone ring, which opens the ring resulting in the loss of the planar structure and potentially its activities. To generate more stable and potent compounds, we synthesized non-hydrolyzable cyclic ether derivative, UAS03 (6*H-*benzo[c]chromene-3,8-diol) (Fig. 1a). The stability of both compounds was examined under conditions of gastric pH and digestive enzymes. The results showed that UAS03 indeed is stable at gastric pH and also in the presence of gastric enzymes e.g., esterases and proteases (Fig. 1a). Both UroA and UAS03 significantly decreased LPS induced IL-6 and TNF-α in mouse bone marrow derived macrophages (BMDMs) with UAS03 showing anti-inflammatory activities even at nano molar concentrations (Fig. 1b). Next, anti-inflammatory activities of UroA and UAS03 were examined in vivo in a LPS-induced peritonitis mouse model. UroA or UAS03 treatment significantly reduced the LPS-induced increase in serum IL-6 and TNF-α levels (Fig. 1c). These results suggest that UAS03 is a potent structural analogue of UroA with increased anti-inflammatory activities.

### UroA/UAS03 induce tight junction proteins

Since, microbial metabolites are in close proximity to gut epithelium; we surmise that metabolites could have a direct impact on epithelial cell function. To examine such effects, we performed RNA-Seq analysis of epithelial cell line (HT29) exposed to UroA. The analysis was performed as described in methods and to determine significance of differential gene expression, cuffdiff2 algorithm was used. Based on an uncorrected *p*-value cutoff of 0.05, 1960 genes were determined to be significantly differentially expressed as a result of UroA treatment in HT29 cells. Further restricting this list, 437 genes were found to be differentially expressed at FDR corrected *q* value <0.05 in UroA treated HT29 cells (Supplementary Fig. 1 and Supplementary Data 1). The pathway analysis using this restricted gene list was performed using Ingenuity Pathway Analysis (IPA) software (Supplementary Fig. 1). The Eukaryotic Initiation Factor 2 (eIF2), mammalian target of rapamycin (mTOR) and mitochondrial dysfunction pathways were emerged as top 3 pathways. The impact of UroA on mitochondrial dysfunction (pathways of mitophagy) have been described in previously by Ryu D et al.^22^. They demonstrated that UroA induced mitophagy and prolonged the lifespan of *C*. *elegans* and increased muscle function in rodents. The impact of UroA on mTOR and eIF2 pathways need to be established in the context of colon epithelial functions. The RNA-Seq analysis showed that cytochrome P450 1A1 (*Cyp1A1*) is among the top 3 UroA upregulated genes (Supplementary Data 1). The pathways analysis further indicated that the Nrf2 and AhR signaling pathways are in top 25 (Supplementary Fig. 1). We surmise that regulation of barrier function is of critical importance in mitigating IBDs. Therefore, we examined the expression of the tight junction proteins in RNA-Seq data and found that claudin 4 (*Cldn4*) is upregulated in UroA treated cells (Supplementary Fig. 2)^24^. In addition to *Cldn4* and *Cyp1A1*, UroA also significantly increased the expression of heme oxygenase 1 (*HMOX1 or HO1*) (Supplementary Fig. 2). HO1 is well known Nrf2-dependent gene, which exerts wide variety of beneficial activities including removal of toxic heme, protection against oxidative stress, regulation of apoptosis, and inflammation^25^. Based on these observations, we hypothesized that UroA and UAS03 will induce tight junction proteins and enhance barrier function through AhR and Nrf2 pathways

Ingenuity Pathway Analysis (IPA) revealed significant enrichment of Nrf2 and AhR signaling pathways (Supplementary Fig. 1), supporting a role for these pathways in UroA signaling. A potential therapeutic avenue in IBD is the ability to increase barrier function. It was therefore of interest that we observed a significant increase in expression of the tight junction protein Cldn4 in UroA treated cells. Although not statistically significant in our RNA-seq dataset, we further observed an increase in expression of additional tight junction proteins ZO-1 and Ocln1 using real-time PCR (Fig. 1d). The increased levels of these proteins by UroA or UAS03 was confirmed by western blots (Fig. 1e) and Cldn4 by confocal imaging (Fig. 1f) in both HT29 and another colon epithelial cell line, Caco2. Further, we observed elevated expression of Cldn4 in the colons of mice treated with UroA/UAS03 (Supplementary Fig. 3a). The functional consequence of increased tight junction proteins was examined using in vitro FITC-dextran permeability assay in transwell plates. As shown in Fig. 1g, pretreatment of Caco2 or HT29 cells with UAS03 or UroA significantly inhibited LPS induced leakage of FITC-dextran into bottom chambers. Overall, these results suggest that treatment with UroA/UAS03 increased the expression of tight junction proteins potentially enhancing the gut barrier integrity.

### AhR mediates the activities of UroA/UAS03

**Fig. 2:**
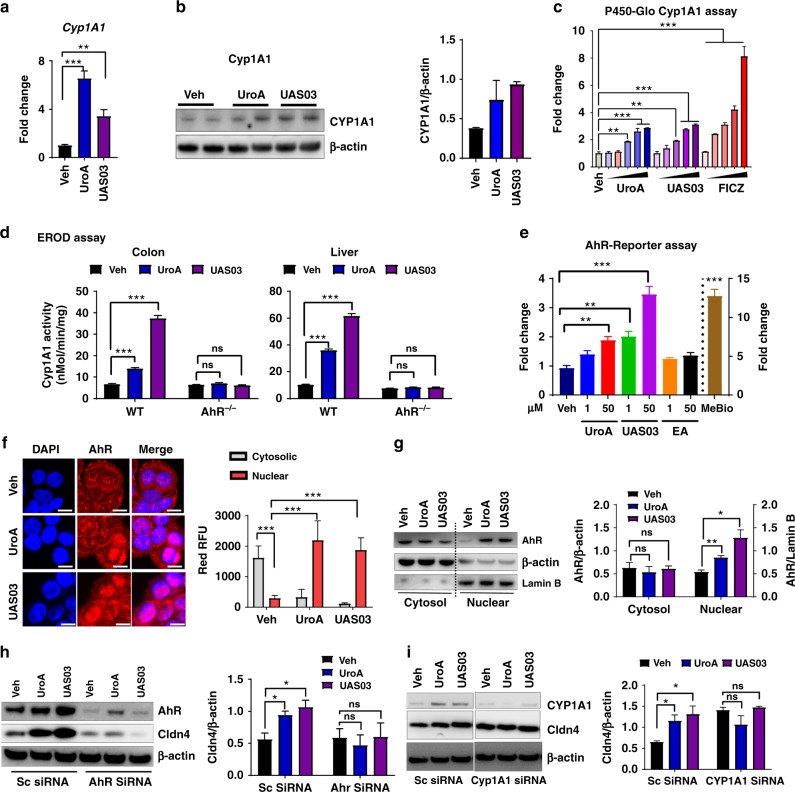
UroA/UAS03 enhance tight junction proteins in AhR-dependent manner. **a** HT29 cells were treated with vehicle (DMSO-0.01%)/UroA/UAS03 (50 μM) for 24 h. mRNA levels of Cytochrome P450 1A1 (*Cyp1A1*) was measured by RT PCR. **b** Cyp1A1 protein levels were measured using immunoblots and quantified band intensities by Image J software. **c** Cyp1A1 enzyme activity was measured by P450-Glo Cyp1A1 assay. HT29 cells were treated with UroA or UAS03 (0.1, 1, 10, 25, 50 µM) or FICZ (0.1, 1, 10, 25, 50 nM) for 24 h and enzyme Cyp1A1 activity was measured. **d** C57BL/6 and AhR^−/−^ (*n* = 3) mice were treated orally with Vehicle (0.25% CMC), UroA or UAS03 (20 mg/kg) for 1 week and Cyp1A1 activity was measured in colons and livers by ethoxyresorufin-O-deethylase (EROD) assay. **e** The cells expressing AhR-reporter (luciferase) were treated with Veh or UroA/UAS03 or ellagic acid (EA) or MeBio (AhR high affinity ligand) for 6 h and fold change of luminescence over vehicle treatment was measured. **f** Immunofluorescence confocal images of HT29 cells treated with vehicle/UroA/UAS03 (50 μM) for 6 h. The cells were stained with anti-AhR antibody (red) and DAPI (blue). Relative fluorescence (*n* = ~20 cells) in the cytosol and nucleus was measured. The scale bar indicates 10 μm. **g** AhR levels in cytosol and nuclear fractions of HT29 cells treated for 2 h with Veh or UroA/UAS03 (50 μM). **h** AhR or **i** Cyp1A1 was knocked down using siRNA in HT29 cells and the cells were treated with vehicle/UroA/UAS03 (50 μM) for 24 h and immnunoblots were performed to detect expression of AhR, Cyp1A1, and Cldn4. Scrambled (Sc) siRNA transfections were used as controls. Immunoblots were quantified using Image J software. The data is representative of two independent repeats with triplicate wells for each treatment. Statistics performed using unpaired *t*-test using Graphpad Prism software. All in vitro studies were performed in triplicates. Error bars, ±SEM; ***p < 0.001; **p < 0.01; **p < 0.05. Source Data are provided as a Source Data File

RNA-Seq data and real-time PCR data suggested that UroA significantly upregulated *Cyp1A1* (Supplementary Data 1, Supplementary Fig. 2, Fig. 2a, b). The P450-Glo Cyp1A1 assay (Fig. 2c) as well as 7-ethoxyresorufin-*O*-deethylase (EROD) assay (Supplementary Fig. 4) were performed to determine, whether the Cyp1A1 enzyme activity was similarly affected. UroA/UAS03 significantly induced Cyp1A1 activity in colon epithelial cells (Fig. 2c and Supplementary Fig. 4). Since Cyp1A1 is a well-known downstream target of AhR signaling^26^, we examined whether UroA/UAS03 mediate their actions through AhR. In these assays, we utilized well established potent AhR ligands [2,3,7,8-tetrachlorodibenzo-p-dioxin (TCDD) or 6-Formylindolo[3,2-b]carbazole (FICZ) and low affinity AhR ligand (beta-naphthoflavone (BNF)] to compare the Cyp1A1 activities with UroA/UAS03. UroA/UAS03 activated Cyp1A1 similar to low affinity AhR ligand BNF at 50 µM. As expected, the high affinity ligands such as FICZ and TCDD showed increased Cyp1A1 activity even at nano molar concentrations compared to UroA/UAS03/BNF (Fig. 2c and Supplementary Fig. 4). More importantly, we tested whether UroA/UAS03 induce the Cyp1A1 activities in vivo using wild type and AhR^−/−^ mice. As shown in Fig. 2d, UroA/UAS03 significantly activated Cyp1A1 activity in colon and liver of wild type but not in AhR^−/−^ mice. Moreover, UroA/UAS03 treated wild type mice showed relatively more Cyp1A1 activity in colon tissues compared to BNF and FICZ treated mice (Supplementary Fig. 5a, b). Interestingly, FICZ and BNF that are delivered through intra peritoneum (i.p) showed more Cyp1A1 activity in liver compared to UroA/UAS03 that are delivered through oral route (Supplementary Fig. 5a, b). It could be attributed to first pass effect. To directly compare the administration route, we delivered UroA or UAS03 or FICZ through i.p. and determined Cyp1A1 enzyme activities. As expected, high affinity AhR ligand, FICZ, induced Cyp1A1 activity ~30 fold in liver compared to 5–6 fold by UroA/UAS03 (Supplementary Fig. 5c). In colons, FICZ only increased Cyp1A1 activity up to ~5 fold, whereas UroA/USA03 increased by only ~3 fold. In summary, these results suggest that UroA/UAS03 upregulate expression of Cyp1A1 and enhances the enzyme activity through AhR albeit at low levels in vivo.

The direct activation of AhR by UroA/UAS03 was examined in HT29 cells by XRE-luciferase reporter assay as well as nuclear translocation of AhR. The data showed that UroA/UAS03 treatment resulted in 2–4 fold induction of luciferase activity (Fig. 2e) compared to the high affinity ligand MeBio that caused higher levels (~15 fold) of AhR activation. Both UroA and UAS03 induced the nuclear translocation of AhR (Fig. 2f, g). AhR was upregulated in mice treated with UroA or UAS03 (Supplementary Fig. 3b). Next, we asked whether AhR or Cyp1A1 are required for UroA/UAS03 mediated upregulation of tight junction protein, Cldn4. For this purpose, *AhR* or *Cyp1A1* expression was suppressed using siRNA knockdown and Cldn4 expression was examined. As shown in Fig. 2h, i and Supplementary Fig. 6, UroA/UAS03 failed to induce Cldn4 both in AhR or Cyp1A1 knockdown cells. In addition, we also deleted Cyp1A1 in HT29 cells using CRISPR/Cas9 methods and examined UroA/UAS03 mediated activities. Deletion of Cyp1A1 did not show effect on basal levels of Cldn4 compared to parental HT29 cells (Supplementary Fig. 7a). As shown in Supplementary Fig. 7b–d, UroA/UAS03 failed to upregulate Cldn4 or NQO1 in Cyp1A1 deleted cells. These results suggest that UroA/US03 induce the expression of tight junction proteins through activation of AhR-Cyp1A1-dependent pathway.

### UroA/UAS03 enhance gut barrier function through Nrf2

**Fig. 3:**
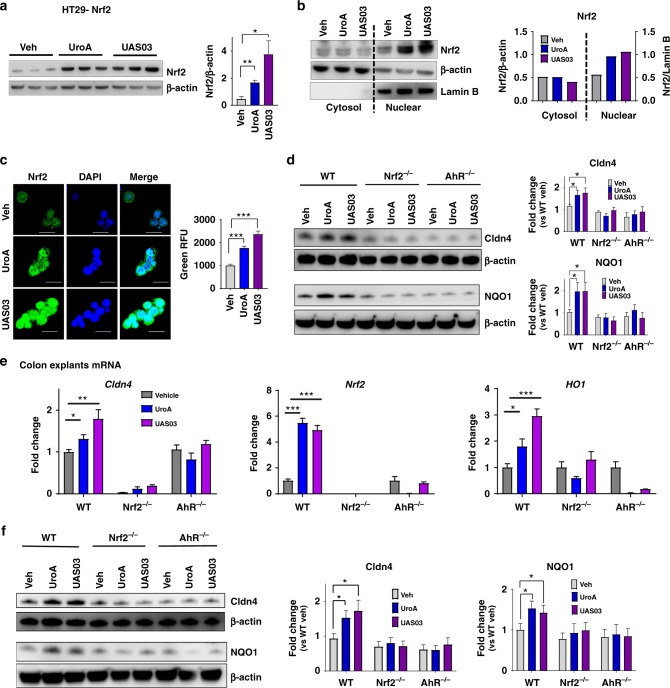
Nrf2 is required for UroA/UAS03 mediated upregulation of tight junction proteins. **a** Nrf2 levels were determined by immunoblots in HT29 cells treated with vehicle/UroA/UAS03 (50 μM) for 24 h. **b** Nrf2 expression in cytosolic and nuclear fractions of HT29 cells treated with Veh/UroA/UAS03 (50 μM) for 6 h. **c** Immunofluorescence confocal images of HT29 cells treated with vehicle/UroA/UAS03 (50 μM) for 6 h. The cells were stained with anti-Nrf2 antibody and DAPI. Relative green fluorescence (*n* = ~20 cells) intensity was measured. Scale bars indicate 25 μm. **d** Expression of Cldn4 and NQO1 in colon explants from WT, Nrf2^−/−^, and AhR^−/−^ mice treated with vehicle/UroA/UAS03 (50 μM) for 24 h. Immunoblots were quantified using Image J software. **e** mRNA levels of *Cldn4, Nrf2*, and *HO1* from colon explant cultures was measured by real-time PCR using SyBr green method. **f** C57BL/6, Nrf2^−/−^, and AhR^−/−^ mice (*n* = 3) treated orally daily with veh or UroA/UAS03 (20 mg/kg) for 1 week. Cldn4 and NQO1 protein levels in colons were measured by immunoblots and quantified by Image J software. All in vitro studies were performed in triplicates. The immunoblots of colon explants and colon tissues were quantified from at least 6 independent runs. The levels of proteins were normalized to β-actin and Wild type vehicle treatment was set to 1 and calculated the fold changes. Statistics performed using unpaired *t*-test using Graphpad Prism software. Error bars, ±SEM; *p < 0.05; **p < 0.01; ***p < 0.001. Source Data are provided as a Source Data File

Since AhR is required for UroA mediated activities, we analyzed existing AhR-ligand Chip analysis using ChIP-Atlas (http://chip-atlas.org/target_genes) that were performed on breast cancer cell line MCF-7 (http://dbarchive.biosciencedbc.jp/kyushu-u/hg19/target/AHR.1.html). The analysis suggested that Nrf2 is a target of AhR signaling cascade (Supplementary Fig. 8a). Similarly, AhR also has influence on tight junction proteins such as Ocln, TJP3, Cldn2, 3 and 5 (Supplementary Fig. 8b). Furthermore, the pathway analysis of our RNA seq data (Ingenuity) also revealed that AhR and Nrf2 pathways are listed in top 25 (Supplementary Fig. 1). Previously, it was shown that TCDD mediates some of its activities through Nrf2 pathways^27,28^. Therefore, we hypothesized that UroA/UAS03 induce tight junction proteins through activating AhR-Nrf2 dependent pathways. We tested this hypothesis in colon epithelial cells as well as in mice deficient in AhR and Nrf2. Treatment with UroA/UAS03 significantly upregulated *Nrf2* both at mRNA and protein levels (Supplementary Fig. 9a and Fig. 3a) and induced its nuclear translocation in HT29 cells (Fig. 3b, c). Nrf2-promoter activities were validated utilizing ARE-luciferase assays, where UroA/UAS03 significantly enhanced luminescence upon treatment (Supplementary Fig. 9b) similar to known Nrf2 activator sulforaphane (SFN) albeit at lower levels. Nrf2 and its target gene HO1 are upregulated in the colons of wild type mice treated with UroA/ UAS03 (Supplementary Fig. 3) as well as in HT29 cells (Supplementary Fig. 9c). To examine the precise function and interdependency of AhR-Nrf2 pathways in UroA/UAS03 induced Cldn4 upregulation, we utilized colon explants from C57BL/6 (wild type, WT), AhR^−/−^ and Nrf2^−/−^ mice. NAD(P)H:quinone oxidoreductase (NQO1) encodes cytoplasmic 2-electron reductase and the induction is shared by both AhR and Nrf2 pathways^27^. We examined whether UroA/UAS03 upregulate expression of NQO1 in colon explants of these mice. Treatment with UroA/UAS03 induced the expression of both Nrf2, NQO1, and Cldn4 in WT colon explants (Fig. 3d, e and Supplementary Fig. 10). But these compounds failed to induce Cldn4 and NQO1 in both Nrf2^−/−^ and AhR^−/−^ colon explants as well as Nrf2 in AhR^−/−^ mice colon explants (Fig. 3d, e, Supplementary Fig. 10) suggesting requirement of AhR and Nrf2 expression for UroA/UAS03 mediated activities. The basal level comparison of expression of Cldn4 and NQO1 in WT, AhR^−/−^, and Nrf2^−/−^ mice colon explants suggests that lack of AhR and Nrf2 reduced the expression of NQO1 and Cldn4 (Supplementary Fig. 10a). The data suggest that expression of Clnd4 is reduced in AhR^−/−^ and Nrf2^−/−^ but not in Cyp1A1 knock down cells.

To define the in vivo requirement of AhR and Nrf2 for UroA/UAS03 mediated upregulation of tight junction proteins, we utilized WT, Nrf2^−/−^, and AhR^−/−^ mice. Examination of basal level expression of Cldn4, NQO1 in the colon tissues of these mice suggests that lack of AhR or Nrf2 have reduced significantly NQO1 levels, but did not show statistical significance for reduction of Cldn4 albeit there was a trend towards reduced expression. (Supplementary Fig. 11c). The mice were treated daily with UroA/UAS03 (orally, 20 mg/kg bodyweight) for 7 days and barrier functions were analyzed. Treatment with UroA/UAS03 significantly upregulated Nrf2 and tight junction proteins (Cldn4, NQO1, Ocln, ZO1, and TJP3) in WT mice (Fig. 3f and Supplementary Fig. 11). In contrast, UroA/UAS03 failed to induce these proteins in Nrf2^−/−^ and AhR^−/−^ mice (Fig. 3f and Supplementary Fig. 11). UroA/UAS03 induced NQO1 expression was also confirmed in HT29 cells (Supplementary Fig. 9d). Overall these results suggest that both AhR and Nrf2 are required for UroA/UAS03 mediated upregulation of tight junction proteins and NQO1.

### Treatment with UroA/UAS03 mitigates colitis

**Fig. 4:**
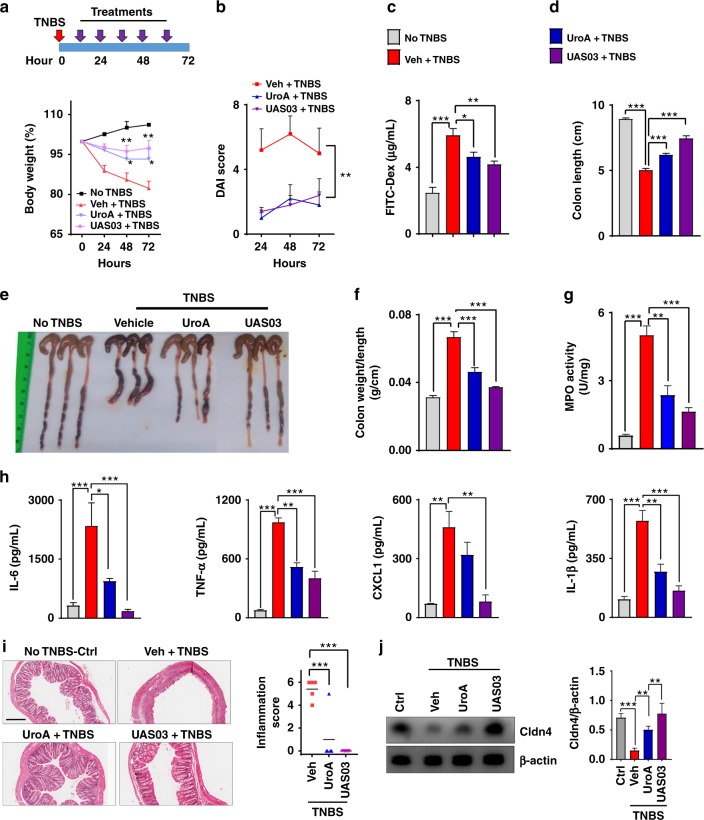
UroA/UAS03 treatment attenuates TNBS-induced colitis in mice. Colitis was induced by intrarectal administration of TNBS (2.5 mg/mouse) in C57BL/6 (8 week age old, *n* = 5/group) mice. Mice were orally treated with vehicle or UroA (20 mg/kg) or UAS03 (20 mg/kg body weight) every 12 h post-TNBS instillation for 60 h and the experiment terminated at 72 h. Representative data from one of three independent experiments is shown. **a** Percent body weight loss (No TNBS- Solid black line; Veh + TNBS- Solid red line; UroA + TNBS- Solid blue line; UAS03 + TNBS- Solid purple line). **b** disease activity index, **c** intestinal permeability, **d** colon lengths were measured. **e** Gross morphological changes of colon, **f** ratio of colon weight/length, **g** colonic myeloperoxidase (MPO) levels, **h** serum IL-6, TNF-α, CXCL1, and IL-1β levels, **i** microphotographs of hematoxylin and eosin (H&E) stained sections of colons and inflammation scores are shown. Scale bar indicates 300 μm. **j** Cldn4 expression in the colons of these mice (*n* = 3) was measured by immunoblots and quantified. Statistical analysis was performed (unpaired *t*-test) using Graphpad Prism software. Error bars, ±SEM ***p < 0.001; **p < 0.01 *p < 0.05. Source Data are provided as a Source Data File

The physiological relevance of UroA/UAS03 regulated barrier function was examined in the 2,4,6-Trinitrobenzenesulfonic acid (TNBS)-induced colitis model^29^. Oral treatment with UroA/UAS03 (20 mg/kg at 12 h intervals) significantly protected from TNBS-induced body weight loss (Fig. 4a), reduced disease activity index (DAI) score (Fig. 4b) and intestinal permeability (Fig. 4c). UroA/UAS03 treatment significantly protected from TNBS-induced colon shortening (Fig. 4d, e) and reduced weight to length ratio (Fig. 4f) suggesting decreased colonic inflammation. UroA/UAS03 treatment also reduced neutrophil infiltration as evident from myeloperoxidase (MPO) activity (Fig. 4g) as well as serum inflammatory markers such as IL-6, TNF-α, CXCL1, and IL-1β (Fig. 4h) that are hallmarks of ulcerative colitis. Consistent with these findings, H&E analysis of colon sections showed significantly less tissue damage and inflammation scores (Fig. 4i). Furthermore, UroA/UAS03 also protected from TNBS-induced downregulation of Cldn4 in the colons of these mice (Fig. 4j). We further examined the effects of dose and frequency of UroA/UAS03 treatments as well as their preventive efficacy in mitigating colitis. As shown in Supplementary Fig. 12, UroA**/**UAS03 mitigated TNBS-induced colitis with a single treatment at 4 or 20 mg/kg body weight. The comparisons bodyweights at each time points suggest that TNBS treatment in all the groups led to decrease in body weight and treatment seems to decrease the loss of body weight, but did not reach significance (Supplementary Fig. 13). However, treatments significantly showed impact on other parameters such as protecting from shortening of colons, blocking inflammatory mediators. Supplementing wild type mice with UroA or UAS03 did not exhibit any signs of toxicity as evident from no observed changes in their body weights, CBC counts as well as serum ALT and AST levels (Supplementary Fig. 14).

**Fig. 5:**
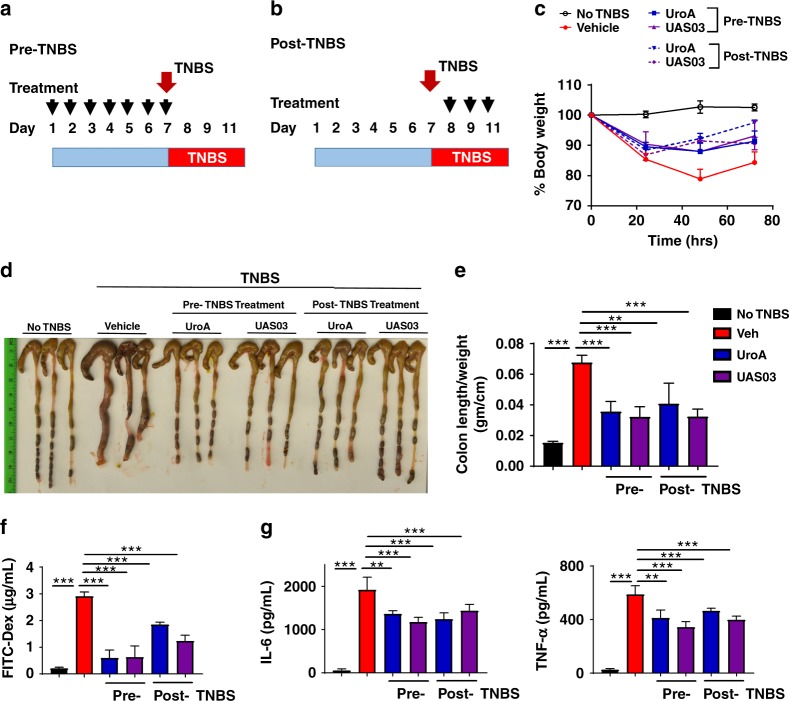
UroA/UAS03 prevent TNBS-induced colitis and sustain beneficial barrier activities. **a** Pre-TNBS treatment. Male C57BL/6 mice (*n* = 5 per group at 7–8 week old age) were given orally vehicle (Veh; 0.25% sodium carboxymethylcellulose) or UroA or UAS03 (20 mg/kg/bodyweight) daily for one week followed by rectal administration of TNBS to induce colitis. These mice did not receive any treatment post-TNBS administration. Mice were euthanized 72 h post-TNBS administration and characterized. **b** Post-TNBS treatment. Another set group of C57BL/6 mice (*n* = 5 per group at 7–8 week old age) received Veh or UroA or UAS03 (20 mg/kg) 24, 48, and 72 h post-TNBS. **c** Percent body weight loss was recorded after TNBS-administration. (No TNBS- Solid black line; Veh + TNBS- Solid red line; Pre-TNBS + UroA- Solid blue line; Pre-TNBS + UAS03- solid purple line; Post-TNBS + UroA- dashed blue line; Post-TNBS + UAS03- dashed purple line). **d** Representative colon images of control (no TNBS) along with vehicle/UroA/UAS03 treated mice from pre- and post-treatment groups. **e** Ratio of colon weight/length, **f** intestinal permeability was evaluated using FITC-dextran leakage assay. **g** Serum levels of IL-6 and TNF-α were measured using standard ELISA methods. Statistical analysis was performed (unpaired *t*-test) using Graphpad Prism software. Error bars, ±SEM ***p < 0.001. Source Data are provided as a Source Data File

Since UroA/UAS03 exhibited strong barrier protective activities by upregulating tight junction proteins, we investigated whether regular exposure to these metabolites would have sustained beneficial effects in preventing colitis. The prophylactic activity profile of UroA/UAS03 was examined in the TNBS-induced colitis model. WT mice were orally fed daily with vehicle or UroA/UAS03 for 1 week followed by TNBS administration to induce colitis. These mice did not receive any further UroA/UAS03. The treatment regimen and percent bodyweights are shown in Fig. 5a and Supplementary Fig. 15. The pre-treated mice were significantly protected from TNBS-induced colon shortening and colonic inflammation (colon length/weight) similar to a therapeutic regimen (Fig. 5b–d). Pre-treatment also significantly enhanced barrier function and decreased TNBS-induced inflammation (Fig. 5e, f). These results suggest that UroA/UAS03 mediated enhanced gut barrier function will likely have long-term beneficial effects in preventing colitis. In therapeutic regimen, mice were treated with UroA or UAS03 24 h post-TNBS, where mice develop severe colitis. In this setting, treatment with UroA/UAS03 also significantly reversed the colitis phenotype by reducing shortening of colons, gut permeability and inflammation compared to vehicle treatment.

**Fig. 6:**
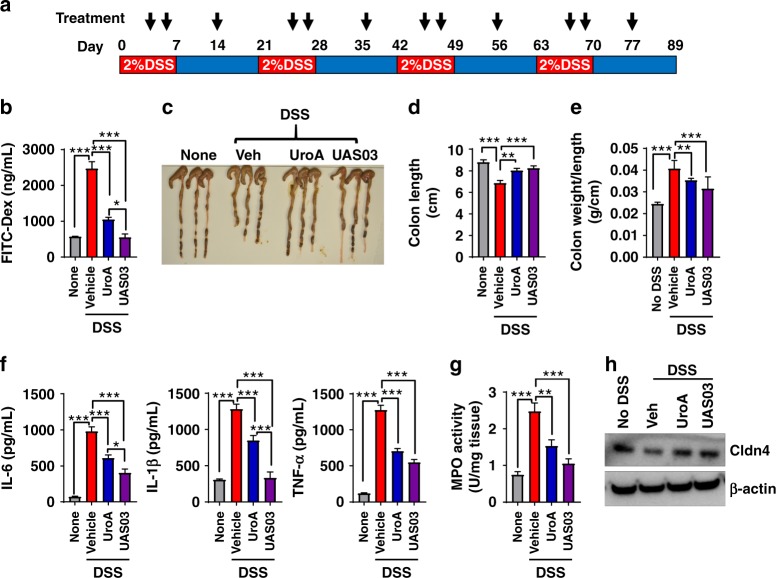
Treatment with UroA/UAS03 mitigate DSS-induced chronic colitis. **a** C57BL/6 mice (7–8 week age old) were treated with four cycles of DSS (2%) with 7 days/cycle with an interval of 14 days with regular water. Control group of mice (*n* = 5) received the regular water without DSS. UroA/UAS03 (20 mg/kg/day/body weight) that was resuspended in 0.25% sodium carboxymethylcellulose (CMC) solution (*n* = 9) or vehicle (CMC) (*n* = 9) was administered on 4th and 6th day of each DSS cycle and one treatment while on regular water. *n* = 5/control; *n* = 9/veh and UroA; *n* = 8/UAS03 group) Mice were euthanized at day 89 and the colitis phenotype was characterized. **b** Intestinal permeability using FITC-dextran was evaluated. **c** Representative colon images **d** colon lengths, **e** ratios of colon weight/length are shown. **f** Serum levels of IL-6, IL-1β, and TNF-α were measured using ELISA methods. **g** MPO levels were determined in colon tissues. **h** Cldn4 expression in the colons of these mice (*n* = 3) was measured by immunoblots. Statistics performed using unpaired *t*-test using Graphpad Prism software. Error bars, ±SEM ***p < 0.001; **p < 0.01; *p < 0.05. Source Data are provided as a Source Data File

The therapeutic applications of UroA/UAS03 were also examined in the dextran sodium sulfate (DSS)-induced colitis model. DSS chemically disrupts the epithelial cell barrier and leads to increased penetration of bacteria resulting in inflammation and colonic tissue damage. As shown in Supplementary Fig. 1^6^, the mice treated with UroA/UAS03 were significantly protected from 3% DSS induced acute colitis. UroA/UAS03 treatment mice displayed overall decreased DAI scores during the disease progression. Importantly, UroA/UAS03 treatments protected from shortening of colons, decreased gut permeability and reduced inflammation compared to vehicle treatment (Supplementary Fig. 16) at the end of experiment on day 15. Further, the therapeutic efficacies of UroA/UAS03 were also examined in a chronic DSS model, where mice were given 4 cycles of 2% DSS in drinking water for 7 days with an interval of 14 days in each cycle on regular water (Fig. 6a). Treatment with UroA/UAS03 significantly protected from DSS-induced colitis as evident from decreased gut permeability (Fig. 6b), reduced shortening of colons (Fig. 6c, d), increased colon weight/length ratio (Fig. 6e), reduced inflammation (serum IL-6, IL-1β, TNF-α as well as colonic tissue MPO levels) (Fig. 6f, g). Analysis of tight junction proteins in these mice also suggest that treatment with UroA/UAS03 enhanced the expression of Cldn4 (Fig. 6h). These results highlight the model independent beneficial activities of UroA/UAS03 in preserving the barrier integrity and mitigating colonic inflammation.

### UAS03/UroA mediated protection against colitis requires AhR-Nrf2 pathways

**Fig. 7:**
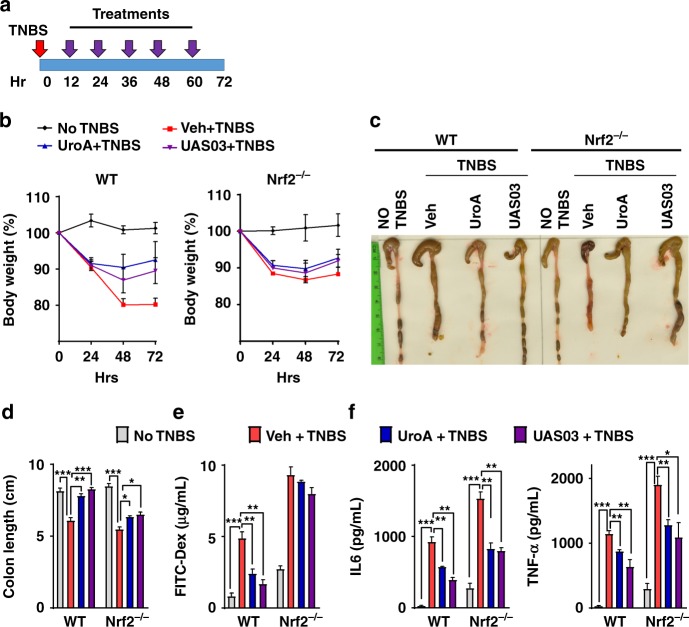
UroA/UAS03 utilize Nrf2 pathways to mitigate colitis. **a–e** Colitis was induced using TNBS in C57BL/6 (WT) and Nrf2^−/−^ mice (*n* = 4–5/group 7–8 week old age). Mice were treated with Veh or UroA/UAS03 (20 mg/kg bodyweight) every 12 h post TNBS administration ending at 72 h. Representative data from two independent experiments is shown. **a** TNBS-induced colitis experimental design and treatment regimen. **b** Percent body weight loss (No TNBS- Solid black line; Veh + TNBS- Solid red line; UroA + TNBS- Solid blue line; UAS03 + TNBS- Solid purple line), **c** representative colon images, **d** colon lengths, **e** gut permeability, **f** serum levels of IL-6 and TNF-α were determined. Statistical analysis was performed (unpaired *t*-test) using Graphpad Prism software. Error bars, ±SEM ***p < 0.001; **p < 0.01 *p < 0.05. Source Data are provided as a Source Data File

**Fig. 8:**
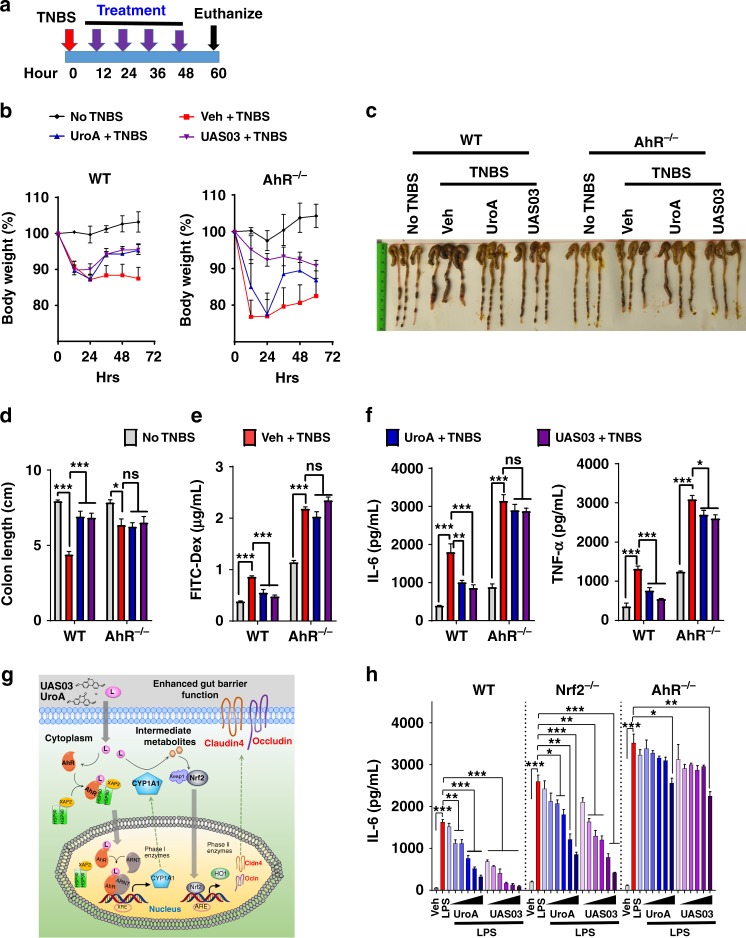
UroA/UAS03 exert beneficial activities through AhR-dependent pathways. **a**–**e** Colitis was induced using TNBS in C57BL/6 (WT) and AhR^−/−^ mice (*n* = 4/group 7–8 week old age). Mice were treated with Veh or UroA/UAS03 (20 mg/kg bodyweight) every 12 h post TNBS administration and mice were euthanized at post 60 h TNBS administration. **a** TNBS-induced colitis experimental design and treatment regimen. **b** Percent body weight loss (No TNBS- Solid black line; Veh + TNBS- Solid red line; UroA + TNBS- Solid blue line; UAS03 + TNBS- Solid purple line), **c** representative colon images, **d** colon lengths, **e** gut permeability, **f** serum levels of IL-6 and TNF-α were determined. Statistical analysis was performed (unpaired *t*-test) using Graphpad Prism software. Error bars, ± SEM ***p < 0.001; **p < 0.01; *p < 0.05. **g** AhR-Nrf2 dependent tight junction protein regulation by UroA/UAS03. UroA/UAS03 (L:ligands) bind to AhR and activate its nuclear translocation to induce expression of Cyp1A1 and Nrf2. Further, UroA/UAS03 causes Nrf2-dependent upregulation of tight junction proteins and enhanced barrier function. **h** LPS (50 ng/ml)-induced IL-6 levels were measured in the presence of Vehicle or UroA or UAS03 (0.1, 1, 10, 20, 30, and 50 μM) in bone marrow derived macrophages (BMDM) from wild type (WT), Nrf2^−/−^ and AhR^−/−^ mice. The data is representative of two independent experiments with triplicates. Statistical analysis was performed (unpaired *t*-test) using Graphpad Prism software. Error bars, ±SEM ***p < 0.001; **p < 0.01 *p < 0.05. Source Data are provided as a Source Data File

The studies described above indicated the importance of AhR-Nrf2 pathway in UroA/UAS03 enhanced barrier function. To examine the relevance of these pathways in colitis, we tested the in vivo requirement for Nrf2 (Fig. 7) and AhR (Fig. 8). Treatment of Nrf2^−/−^ mice with UroA/UAS03 failed to restore body weight loss caused by TNBS-induced colitis (Fig. 7a, b and Supplementary Fig. 17) or protect from shortening of colons (Fig. 7c). UroA/UAS03 treatment did not enhance barrier function in Nrf2^−/−^ mice as evident from similar FITC-dextran leakage in UroA/UAS03 treated mice compared to vehicle treatment (Fig. 7d). These results demonstrated that UroA/UAS03 enhanced gut barrier integrity requires the expression of Nrf2. Interestingly, UroA/UAS03 partially reduced serum inflammatory mediators such as IL-6 and TNF-α levels in Nrf2^−/−^ mice (Fig. 7e), suggesting that UroA/UAS03 could mediate some of the anti-inflammatory activities in Nrf2-independent manner. To define the role of AhR in UroA/UAS03 mediated protective activities, the TNBS-induced colitis model was executed in AhR^−/−^ mice along with wild type mice (Fig. 8a). As expected AhR^−/−^ mice were more susceptible to TNBS-induced colitis model as evident from rapid loss of body weight (Fig. 8b and Supplementary Fig. 18). Therefore, we terminated the experiment at post 60 h TNBS administration (Fig. 8a). Treatment with UroA/UAS03 failed to protect from shortening of colon lengths in AhR^−/−^ mice compared to wild type mice (Fig. 8c, d). Additionally, UroA/UAS03 failed to correct the barrier dysfunction in AhR^−/−^ mice as evident from in vivo permeability assays (Fig. 8e). Analysis of serum inflammatory mediators suggest that UroA/UAS03 failed to reduce IL-6 and slightly reduced the TNF-α in AhR^−/−^ mice, whereas UroA/UAS03 treatments significantly reduced IL-6 and TNF-α in wild-type mice as observed above (Fig. 8f). Based on these results we propose that UroA/UAS03 exert protective barrier functional activities through AhR-Nrf2-dependent pathways by inducing tight junction proteins (Fig. 8g).

Since the macrophages are critical mediators of colonic inflammation in IBDs^30,31^, we determined if UroA/UAS03 mediated anti-inflammatory activities require the AhR-Nrf2 pathways in macrophages. First, we examined whether UroA/UAS03 activates Nrf2-dependent pathways in macrophages. The results showed that treatment with UroA/UAS03 significantly upregulated Nrf2 expression and induced its nuclear translocation, as well as upregulation of Nrf2-target genes such as HO1 expression in macrophages (Supplementary Fig. 19a–e). Further, analysis of UroA/UAS03 mediated down regulation of LPS-induced IL-6 production in macrophages from WT, Nrf2^−/−^ and AhR^−/−^ mice showed that LPS-induces much higher levels of IL-6 in Nrf2^−/−^ and AhR^−/−^ macrophages relative to WT (Fig. 8h). UroA/UAS03 also reduced the NF-κB activation in an AhR-dependent manner in macrophages (Supplementary Fig. 19f). AhR^−/−^ BMDM are hyper responsive to LPS stimulation as evident from increased NF-κB activation as well as increased levels of IL-6 compared to wild type (Fig. 8h and Supplementary Fig. 19g). Despite significant lowering of IL-6 levels by UroA/UAS03 in Nrf2^−/−^ macrophages, these reduced levels are still higher compared to LPS-induced IL-6 in WT macrophages. When compared, the fold reduction upon treatments (Supplementary Fig. 20), UroA/UAS03 reduced IL-6 in Nrf2^−/−^ similar to WT indicating Nrf2-independent anti-inflammatory activities both in vivo (TNBS model) and in vitro BMDM (LPS-induced IL-6). In contrast, UroA/UAS03 did not block LPS-induced IL-6 production in AhR^−/−^ macrophages up to 30 μM as well as in AhR^−/−^ mice in TNBS-induced colitis model suggesting that UroA/UAS03 mediate anti-inflammatory activities through AhR-dependent manner. AhR^−/−^ BMDM slight decrease in IL-6 levels at 50 μM dose may suggest some of unknown AhR-independent anti-inflammatory activities. The results presented here highlight that single microbial metabolite regulates the barrier function in epithelial cells via the activating AhR-Nrf2 signaling pathways and also anti-inflammatory activities in AhR dependent pathways.

## Discussion

In this study, we identified that microbial metabolite UroA, and its analogue UAS03, increases overall gut health by enhancing barrier function in addition to their anti-inflammatory activities. UroA/UAS03 activate the phase I (AhR-Cyp1A1) and phase II (Nrf2- anti-oxidative pathways) metabolic pathways to enhance expression of tight junction proteins and inhibit inflammation. We further demonstrate that treatment with these compounds significantly mitigated colitis both in preventive and therapeutic settings.

A key physiological function of gut microbiota is to catabolize dietary components into absorbable metabolites. Despite the identification of numerous microbial metabolites, the molecular targets and mechanisms of action for many metabolites is unknown. Urolithins, derived from dietary polyphenols such as ETs, EA by microbiota are linked to the beneficial effects associated with high consumption of fruits and vegetables in humans^11,18,19^. It was reported that the *Bifidobacterium pseudocatenulatum INIA P815* strain was able to metabolize EA to produce UroA and UroB^32^. Large inter-individual variability in UroA levels^18^ suggests that bacteria responsible for UroA production may also be highly variable in humans. Urolithin levels can reach up to micro molar concentrations in human serum depending on their microbiota composition^18^. The premise of this study is that direct supplementation of UroA will overcome the intrinsic variation in microbiota among populations and offer health benefits. In this regard, we also successfully developed UAS03, a more stable and potent structural analogue of UroA that displayed increased gut barrier protection and anti-inflammatory activities.

Previous studies demonstrated inhibitory activities of urolithins in inflammation, proliferation, and aging in various models^20,22,33^. However, the molecular targets or mechanisms of action of these metabolites on pathophysiological processes are unknown. Our approach of searching for an epithelial cell function for these metabolites by RNA-Seq analysis revealed several important clues for their function and potential mechanisms. UroA/UAS03 mediated up regulation of tight junction proteins (e.g., Cldn4, Ocln, and ZO1) and protection from LPS induced leakage in epithelial monolayers showed that these metabolites clearly play an important role in the regulation of barrier function. Tight junctions consist of both transmembrane proteins (e.g., occludin, claudins, junctional adhesion molecules, and tricellulin) as well as peripheral membrane proteins (e.g., ZO-1 and cingulin) to regulate paracellular permeability and maintain gut barrier function. The disruption of tight junctions leads to barrier dysfunction and is implicated in IBDs and other disorders^34^. In particular, gut barrier dysfunction leads to bacterial invasion and excessive inflammation^35,36^. The inflammatory cytokines and growth factors such as TNF-α, IFNγ, IL-1β, TGF-α, and platelet derived growth factors as well as bacterial endotoxins (LPS) are known to increase permeability by disrupting tight junctions^37^. Thus, barrier dysfunction and inflammation form a self-perpetuating loop in IBDs and blocking one of these is often insufficient for mitigating the disease process.

Our RNA-seq studies and expression analysis showed that in addition to upregulation of *Cldn4*, UroA also induced the expression of *Cyp1A1* and *HO1* in colon epithelial cells. Since *Cyp1A1* and *HO1* represent the activation of phase I and phase II drug metabolic pathways, these results suggested the potential involvement of AhR and Nrf-2 in mediating UroA/UAS03 functions. AhR is a nuclear transcription factor that responds to both xenobiotic and endogenous ligands leading to cell-specific gene regulation and cellular functions. AhR activation is responsible for the induction of multiple Phase I and Phase II xenobiotic chemical metabolizing enzymes such as Cyp1A1^38^. AhR can be activated by many chemicals including environmental polycyclic aromatic hydrocarbons, coal tar, phytochemicals, and products from commensal bacteria and tryptophan metabolism^39^. Historically, human exposure to high affinity AhR ligand, TCDD (which is not metabolized by Cyp1A1) displayed severe adverse events such as appearance of cysts, eruptions, pustules, and erythema as well as life threatening manifestations including liver, renal failures, myocardial degeneration^39^. However, AhR activation by FICZ ligand (amenable to Cyp1A1 drug metabolism) has been implicated in controlling the immune system and protection from colitis^40,41^. Our studies revealed that UroA/UAS03 treatments induced the expression and nuclear translocation of AhR and enhanced transcription of XRE-target genes as well as induced Cyp1A1 enzyme activities without exhibiting toxicity.

Interestingly, UroA/UAS03 failed to induce Cldn4/NQO1 in Cyp1A1 knockdown cells. Previously, it was reported that overexpression of Cyp1A1 in mice resulted in depletion of natural AhR ligands and deletion of Cyp1-enzymes in mice (Cyp1A1, Cyp1A2, Cyp1B1 triple knockout mice) led to increase in availability of AhR ligands and increased their activities^42^. Similarly, we also anticipated that deletion of drug metabolizing and detoxifying enzyme, Cyp1A1, would enhance the availability of UroA/UAS03 and enhance their activities. However, we did not observe increased activities upon deletion of Cyp1A1 in colon epithelial cells. We still do not understand the complete mechanisms for these observations. It may be necessary to delete all Cyp enzymes to avoid compensation mechanisms to detect increased activities. It also is possible that UroA/UAS03 undergo Cyp1A1 drug metabolism and generates unknown active phase I metabolites, which could activate Nrf2 pathways. It was reported by Gimenez-Bastida et al.^43^ that UroA-glucuronide (UroA-Gluc) forms displayed beneficial activities, where treatment with UroA-Gluc ameliorated the TNF-α induced inflammation and associated molecular markers in human aortic endothelial cells. UroA is known to circulate as glucuronide and sulfate conjugates as well as parent form (UroA) in plasma^20^. Therefore, it is possible that products from UroA drug metabolism Phase I and II intermediates could also exert certain beneficial activities. Alternatively, UroA/UAS03 could induce basal ROS that is dependent on expression of Cyp1A1 and leading to activation of Nrf2-pathways. Additional studies are required to support these possibilities and to define precise role of Cyp1A1 in UroA/UAS03 mediated activities using Cyp1-enzyme whole body and intestinal epithelial cells (villin*)* specific knock out mice.

UroA/USA03 failed to exert their activities in cells lacking AhR or in AhR^−/−^ colon explants as well as in AhR^−/−^ mice suggesting a critical role for the AhR pathway in mediating UroA/UAS03 activities. While the regulation of immune cell function by AhR has been previously demonstrated^41,44^, our current studies highlight the importance of this pathway in epithelial cells to regulate tight junction proteins and barrier function.

Previous studies suggested that interdependency of AhR and Nrf2 pathways^28,45,46^. Nrf2, a basic region-leucine zipper transcription factor, protects cells and tissues from oxidative stress by inducing the expression of antioxidant and phase II-enzymes such as glutathione *S*-transferase and NQO1^47^ as well as controlling LPS-induced inflammation^48^. Our studies both in vitro and in vivo suggest that UroA/UAS03 significantly induced the expression of Nrf2 as well as its target genes such as HO1 and NQO1 in colon epithelium. Furthermore, our results also showed that AhR-Cyp1A1-Nrf2 pathways are essential for UroA/UAS03 mediated upregulation of tight junction proteins (Figs. 2 and 3).

Our extensive studies in colitis models revealed that treatment with UroA/UAS03 significantly enhanced tight junction proteins, decreased gut permeability, and reduced local and systemic inflammation leading to attenuation of colitis (Figs. 4–8). Even a single dose of UroA/UAS03 exhibited therapeutic efficacies against TNBS-induced colitis. Importantly, prophylactic benefits of UroA/UAS03 on gut barrier function and prevention of colitis development was observed (Fig. 5). The mice pre-treated with UroA/UAS03 prior to TNBS administration significantly reduced gut permeability (Fig. 5e), which is consistent with increased expression of tight junction proteins. Despite not receiving further treatments post-TNBS administration, these mice were protected from disease development suggesting prophylactic effects of these compounds through enhanced barrier function. Moreover, UroA/UAS03 supplementing daily for 7 days induced expression of AhR, Nrf2, and Cldn4 in the colons of wild-type mice without observable toxicity (Fig. 3f, Supplementary Figs. 3, 11 and 14) suggesting potential translational applications for these compounds. Further, treatment with UroA/UAS03 also mitigated both chronic and acute DSS-induced colitis indicating model independent beneficial activities of these metabolites Fig. 6 and Supplementary Fig. 16). Previously, it was shown that mice lacking AhR or Nrf2 are more susceptible to colitis compared to wild type^40,49^. In contrast to the toxicity associated with the high affinity AhR ligands such as TCDD, UroA/UAS03 are low affinity non-toxic AhR ligand (partial agonist) like BNF that suppressed the pathogenesis of DSS-induced colitis^40^.

It was suggested that Nrf2 protects from colitis through regulation of pro-inflammatory cytokines and induction of phase II detoxifying enzymes. Kobayashi et al.^50^ demonstrated that Nrf2 suppresses inflammation through redox control, by opposing the transcriptional upregulation of proinflammatory cytokine genes and identified Nrf2 as the upstream regulator of cytokine production. Previously, it was also demonstrated that ablation of Nrf2 leads to enhancement of NF-κB activation resulting in increased inflammatory cytokines production^51^ and severe colitis^49^. We also observed increased basal level of inflammatory mediators in Nrf2^−/−^ mice compared to wild-type mice as well as in Nrf2^−/−^ BMDM. Further, addition of LPS significantly upregulated IL-6 in Nrf2^−/−^ BMDM compared to wild-type BMDM as well as in TNBS-induced colitis model. UroA/UAS03 failed to repair TNBS-induced barrier dysfunction and colitis in Nrf2^−/−^ mice (Fig. 7).

The role of AhR in UroA/UAS03 mediated upregulation of tight junction proteins was demonstrated using AhR siRNA, colon explants from AhR^−/−^ mice as well as in vivo treatments in AhR^−/−^ mice. Additionally, UroA/UAS03 failed to mitigate TNBS-induced colitis in mice lacking AhR (Fig. 8). Interestingly, UAS03 seems to have some protective role against rapid body weight loss in AhR^−/−^ mice that are treated with TNBS. It is not clear at this time, why UAS03 exhibits these beneficial effects. However, it did not protect against other parameters such as shortening colon lengths, increased permeability, and increased inflammatory mediators. We acknowledge the inherent problems of AhR^−/−^ mice. AhR^−/−^ mice inherently suffer from variety of organ disorders including a decline in the efficacy of their immune system and high sensitivity to inflammatory stimuli^52^. Previously, it was demonstrated that activation of AhR protects against colitis^41,53^ and AhR^−/−^ mice develop severe colitis compared to wild type mice and display increased inflammatory mediators^54^. Similarly, our studies also showed that increased susceptibility to TNBS-induced colitis in AhR^−/−^ mice compared to wild type. AhR^−/−^ mice exhibited rapid body weight loss leading to termination of the experiment at 60 h. Generally, the endogenous ligands of AhR regulate multiple functions in the body via AhR and maintains the homeostasis in wild type mice. In AhR^−/−^ mice, the endogenous ligands cannot act as bioregulatory molecules due to lack of AhR potentially leading to severe colitis phenotype compared to wild type mice. It is therefore possible that failure of UroA/UAS03 mediated protective activities against colitis in AhR^−/−^ mice may not provide conclusive evidence for AhR role. Further studies are warranted using *Villin Cre AhR floxed mice*, to tease out involvement of AhR in UroA/UAS03 mediated protective activities in colitis models. Despite plethora effects in AhR^−/−^ mice, studies involving colon epithelial cells, siRNA knockdown, AhR^−/−^ colon explant studies as well as BMDM studies reinforces the involvement of AhR pathways in mediating UroA/UAS03 barrier and anti-inflammatory activities. Recent study from Dr. Brigitta Stockinger’s group^55^ also suggests AhR protects from inflammatory damage by maintaining intestinal stem cell homeostasis and barrier integrity supporting our observations that activation of AhR enhances the barrier integrity. In this paper, they demonstrated that AhR promotes barrier function through direct activity on intestinal epithelial cells (IEC) by using mice that lack AhR in IEC (*Villin*^*Cre*^*Ahr*^*fl/fl*^*)*. These mice exhibit decreased expression of Muc2 and increased levels of IL-6 suggesting that AhR role in barrier integrity and inflammation^55^.

The current studies highlight the critical requirement for AhR-Nrf2 in protecting from barrier dysfunction. It is possible that UroA/UAS03 are exerting colitis protective activities by two pronged mechanisms of action. These compounds directly act on immune cells (e.g., macrophages) to prevent LPS/bacterial induced inflammation as well as exhibit anti-oxidative activities through AhR-Nrf2 pathways. Most importantly, these metabolites have direct impact on gut epithelium and gut barrier function by upregulating tight junction proteins. Enhanced barrier function reduces the bacterial leakage in the gut leading to significant reduction in systemic inflammation. To delineate the effects on immune cells versus epithelial cells, further in depth studies involving cell specific deletion of AhR and Nrf2 in transgenic mice using Cre/lox methodologies are required. RNA-Seq pathway analysis as well as studies by Ryu et al.^22^ suggest that UroA plays an important role in regulating mitochondria functions through inducing mitophagy. Several evidences suggest that mitochondrial dysfunction is a major contributor in the pathophysiology of IBD^56^. It was shown that isolated enterocytes from IBD patients have swollen mitochondria with irregular cristae^57^. The intestinal epithelial cells isolated from experimental colitis mice models also exhibited abnormal mitochondrial structures^58^. Therefore, we speculate that in addition to anti-inflammatory and barrier protective activities, UroA/UAS03 may potentially reduce IBD through regulating mitochondrial dysfunction.

Evolutionarily, human-microbiota developed indigenous mechanisms to protect from external challenges. It is possible that excess use of antibiotics and modern dietary trends led to microbial dysbiosis resulting in the elimination of some bacterial populations that are capable of producing beneficial metabolites. More rigorous and systematic studies are required to assess the beneficial advantages of direct consumption of metabolites in humans both in healthy and disease conditions, whether supplementation of metabolites could overcome the dysbiosis. The current study summarizes one such metabolite, UroA and its analogue, UAS03 with activities in mitigating IBDs by enhancing gut barrier function and reducing inflammation. Existing IBD treatments include utilizing anti-TNF-α antibodies to reduce inflammation; here we suggest that enhancing gut barrier functions in addition to inhibiting inflammation might provide better therapeutic options for control of IBDs. Overall, UroA/UAS03 will not only be efficacious in IBD-related diseases but may also have significant translational implications in other disorders involving barrier dysfunction and inflammation such as alcohol liver diseases, neurological disorders, and colon cancers.

## Methods

### Materials

General laboratory chemicals and reagent solutions were purchased from Sigma-Aldrich (St. Louis, MO). ELISA kits for IL-6 and TNF-α were purchased from Bio-legend. ELISA kit for CXCL1 was purchased from R&D systems. All antibodies were purchased from Santacruz unless otherwise specified. LPS was purchased from Sigma Aldrich. Colitis grade DSS (36,000–50,000 M.W) was purchased from MP Bio. UroA was custom synthesized as previously described^23^.

### Mice

C57BL/6 mice were either bred in our animal facility or purchased from Jackson Laboratories. Breeding pairs of Nrf2^−/−^ mice (B6.129x1-Nfe2/2^tm1Ywk/^J, stock # 0170009) were purchased from Jackson Laboratories and bred at U of L animal facility to generate experimental animals. AhR^−/−^ mice (Model# 9166) were purchased from Taconic Laboratories. We utilized the mice at the ages of between 7–9 weeks age old for colitis experiments. Mice were kept in specific pathogen-free (SPF) barrier conditions with temperature-controlled room with alternate 12 h cycles of dark and light. Animals were allowed free excess to feed and water ad libitum. All studies were performed under approved protocols from Institutional Animal Care and Use Committee (IACUC), University of Louisville, Louisville, KY, USA. Source Data for all the bar graphs are provided as a Source Data File.

### Synthetic procedure for synthesis of UAS03

Chemically UroA (3,8-dihydroxy-6H-dibenzo[b,d]pyran-6-one) structure has a bridge ester, lactone, and two hydroxyl on two phenyl rings. UroA has a lactone (cyclic ester) bond that connects two phenyl rings and leads to the planar structure. Gastric pH or digestive enzymes can hydrolyze the lactone bond leading to opening of the ring. This will result in losing the planar structure, becomes propeller structure, and potentially loses its activities. To generate more stable and potent compounds, we have synthesized non-hydrolyzable cyclic ether derivative, UAS03 by the following procedure (Supplementary Fig. 21).

Sodium borohydride (0.165 g, 4.38 mmol) was added to dry THF (10 ml), and the mixture was cooled 10 °C before borontrifluoride etherate (0.80 g, 5.7 mmol) was added drop wise over a period of 1 h. Then 3,8-dihydroxy-6H-benzo[c]chromen-6-one (Uro-A) (0.5 g, 2.19 mmol) in THF (5 ml) was added over a period of 10 min. The mixture was allowed to stir for 5 h at 50 °C. The completion of reaction was monitored by thin layer chromatography (TLC). The reaction was quenched with methanol. 3 N aqueous HCl solution (10 ml) was added, and the mixture was gently heated to 50 °C for 30 min. The reaction mixture was adjusted to neutral with 10% NaOH solution, and the volatiles were evaporated under reduced pressure. The crude product was purified by column chromatography using 50% ethylacetae in Hexane with 60–120 mesh silica gel to get pure 6H-benzo[c]chromene-3,8-diol product.

MS (M+1) = 215.2. ^1^H-NMR (DMSO-d_6_): *δ*: 9.49 (2H, s), 7.51–7.50 (1H, d, J = 6.6 Hz), 7.48–7.47 (1H, d, J = 6.6 Hz), 6.75–6.73 (1H, m), 6.61 (1H, s), 6.48–6.46 (1H, m), 6.32 (1H, s), 4.96 (2H, s). ^13^C-NMR (DMSO-d_6_): *δ*: 158.10, 156.71, 154.93, 131.88, 123.86, 122.79, 121.66, 115.72, 114.89, 111.84, 110.07,103.95, 68.18.

### Cell cultures

Human colon epithelil carcinoma cell lines, HT29 (ATCC # HTB-38^TM^) and Caco2 cells (ATCC # HTB-37^TM^) were maintained in DMEM-high glucose and EMEM-high glucose (Cornings; 10-009CV) respectively, supplemented with 10% fetal bovine serum, 1X penicillin-streptomycin solution (100 U/ml penicillin, and 100 µg/ml streptomycin; Sigma Aldrich) in a humidified atmosphere (5% CO_2_, 95% air, 37 °C). Mouse bone marrow derived macrophages (BMDMs) were isolated and cultured using the following procedure^59^. Briefly, mice were killed by CO_2_ anesthesia, rinsed in 70% ethanol and bone marrow was isolated from tibias and femurs. Bone marrow cells were plated (2 × 10^6^ cell/ml) in DMEM-high glucose (HyClone) supplemented with 10% FBS, 1% glutamine, 1X penicillin-streptomycin solution and 50 ng/mL mouse M-CSF (R&D Systems Inc., Minneapolis, MN) for 7 days for differentiation.

### Measurements of IL-6 and TNF-α levels in BMDM

BMDM were plated in 96 (10,000 cells/well) and 12 wells (0.1 × 10^6^ cells/well) plate for ELISA and RNA isolation. To evaluate the anti-inflammatory properties, BMDMs were stimulated with *E*. *coli*-derived lipopolysaccharides (LPS; O55:B5; Sigma) at 50 ng/mL concentration for six hours alone or in combination with UroA or UAS03 at indicated concentrations (0.01, 0.1, 1, 10, 25, and 50 μM) in quadruplicates. For cytokine production via ELISA, the supernatant was collected and centrifuged at 10,000 × g for 10 min at 4 °C to pellet down any cell and cytokines were quantified using IL-6 and TNF-α specific ELISA kit (Biolegend) following manufacturer’s instruction.

### LPS-induced peritonitis

Male mice (C57BL/6J; 6–8 weeks old) were randomly divided in 3 groups *viz*. vehicle (0.25% sodium carboxymethylcellulose (CMC)), UroA and UAS03. UroA and UAS03 groups received oral gavage of respective compounds (20 mg/kg in 100 μl of volume) at 0, 6, 12, 18, and 24 h. Vehicle group received same volume of CMC at same time. After 24 h, mice were injected intra-peritoneally with LPS (2 mg/kg; Sigma-Aldrich). Post 4 h LPS challenge, mice were killed and blood was collected. The serum was prepared using BD Microtainer separator tubes. The serum samples were analyzed for IL-6 and TNF-α using respective ELISA assay kit (Biolegend).

### Real-time PCR

Total RNA was isolated from cells/tissue using Maxwell® 16 LEV simplyRNA tissue kit (Promega) and reverse transcribed with TaqMan™ Reverse transcription Kit (Applied Biosystems, CA, USA). The transcribed cDNA (after dilution) was mixed with 100 nM gene specific primers (Real time primers LLC) and 1X SYBR green reaction mix (Power SYBR® Green PCR Master Mix; Applied Biosystems, CA, USA). Changes in gene expression was analyzed using CFX96^TM^ Real-Time System (Bio Rad) and fold change in expression was calculated using 2^-ΔΔ^CT method using GAPDH/β-actin as house keeping gene and normalized with untreated control.

### In vitro permeability study

For in vitro cellular permeability studies, Caco2 cells or HT29 cells (2 × 10^4^ cells/cm^2^) were seeded in 24-well Transwell® plates (Cornings; USA), on polyester membrane filters (pore size 0.4 µm, surface area 1.12 cm^2^)^60^. Culture medium was added to both apical and basal chamber and the medium was changed every other day up to 21 days for Caco2 cells or 5–7 days for HT29 cells. For Caco2 cells, transepethelial electrical resistance (TEER) was calculated using EMD Millipore Millicell-ERS2 Volt-Ohm Meter (Millipore). Filters (with cell monolayer) showing more than 600 Ω.cm^2^ were used for permeability study. After cells reach desired confluence (monolayered cells), cells were pre-treated with vehicle (0.01% DMSO) UroA (50 μM) and UAS03 (50 μM) for 24 h. After treatment, monolayer was washed with PBS to remove any residual drug and 200 μL of LPS (50 ng/ml in HBSS) was added to each well and incubated for 2 h. After LPS treatment, the monolayer was washed with PBS twice and 200 μL of FITC-Dextran (FD-4; Sigma Aldrich, USA) solution (1 mg/mL in HBSS) was added. After 2 h, a sample from the basal chamber was withdrawn and FD4 concentration was determined using fluorescence 96-wells plate reader at excitation and emission wavelengths were 480 and 525 nm, respectively.

### RNA sequencing

Total RNA was isolated from HT29 cells treated with vehicle and UroA (50 μM) (*n* = 3) for 24 h and RNA was isolated using Trizol based lysis followed by Qiagen RNeasy kits. The isolated RNA was checked for integrity (RIN>9.5) using the Agilent Bioanalyzer 2100 system (Agilent Technologies, Santa Clara, CA) and quantified using a Qubit fluorometric assay (Thermo Fisher Scientific, Waltham, MA). Poly-A enriched mRNASeq libraries were prepared following Illumina’s TruSeq Stranded mRNA LT library preparation protocol (Illumina Inc., San Diego, CA) using 1 µg of total RNA. All 15 samples were individually barcoded and quantitated with the KAPA Library Quantitation Kit for Illumina Platforms (Kapa Biosystems, Wilmington, MA) in conjunction with an Agilent Bioanalyzer DNA 1000 analysis (Agilent Technologies, Santa Clara, CA) for fragment size determination. The average fragment size was approximately 300 bp. 1.8 pM of the pooled libraries with 1% PhiX spike-in was loaded on one NextSeq 500/550 75 cycle High Output Kit v2 sequencing flow cell and sequenced on the Illumina NextSeq 500 sequencer. The quality of the 1 × 75 bp sequences was checked using FASTQC (version 0.10.1)^61^. Trimming was not necessary with the median quality score above 30 (error probability = 0.001 or 1 base call in 1000 is predicted to be incorrect) across the entire length of the read and the lower quantile above a score of 20 (error probability = 0.01) at the end of the read where there is an expected decrease in quality. The raw reads for each sample were directly aligned to the *Homo sapiens* (hg38) reference genome assembly (hg38.fa) using tophat2 (version 2.0.13)^62^, generating alignment files in bam format. Optional parameters include –no-coverage-search and –library-type fr-firststrand. The human ENSEMBL^63^ transcriptome gtf v82 was used for transcript identification, resulting in 60,903 total genes. Supplementary Table 1 indicates the number of raw reads successfully aligned for each of the samples. On average, 26 million reads were aligned per sample with a mean alignment rate of 97%. Following sequence mapping, differentially expressed genes were determined using tuxedo suite of programs including cuffdiff2 (version 2.2.1)^64,65^ with the optional parameter –library-type fr-firststrand. The RNA-seq data was deposited in gene data base (GEO # GSE113581).

### Immunoblots (western blots)

The total protein lysates were collected either from colon tissue/cells using radioimmunoprecipitation assay (RIPA) buffer (Sigma-Aldrich, USA) and quantified using BCA protein quantification kit (Thermo Scientific) as per instructional manual. Total protein (20–50 μg) of was resolved on NuPAGE^TM^ 4–12% Bis-Tris gel (Novex Life technologies) and transferred to polyvinylidene difluoride membrane (0.22 μm pore; Millipore, USA). After blocking with 5% (w/v) skim milk powder (containing 1X TBS) for 1 h, the membrane was then incubated with respective antibodies at 4 °C overnight (dilutions of respective antibodies is given in Table 1). Next day, respective secondary antibody conjugated with Horseradish peroxidase were probed and the chemiluminescent substrate was used to detect the protein bands (ImageQuant LAS 4000). Densitometry analysis of bands were done using ImageJ software. Anti-bodies for Cldn4, Ocln, Cldn1, Cyp1A1, AhR, HO1, NQO1, Keap1, β-actin, and Lamin B were purchased from Santa Cruz Biotechnologies (USA) and Nrf2 from Novus Biologicals (USA). Source and list of antibodies are provided in Table 1. The uncropped images of important immunoblots are shown in Supplementary Fig. 22.

**Table 1:** List of antibodies used for western blots

Sr no	Antibody	Source	Dilution
1	Nrf2 (NBP1-32822)	Novus Biologicals	1:1000
2	HO-1 (sc-10789)	Santa Cruz Biotechnology	1:500
3	Cldn4 (sc-376643)	Santa Cruz Biotechnology	1:1000
4	ZO-1 (5406)	Cell Signaling Technology	1:2000
5	Ocln (sc-133256)	Santa Cruz Biotechnology	1:1000
6	NQO1 (sc32793)	Santa Cruz Biotechnology	1:1000
7	β-actin (Sc-47778 HRP)	Santa Cruz Biotechnology	1:5000
8	Lamin B (sc-6216)	Santa Cruz Biotechnology	1:100
9	AhR (sc-133088)	Santa Cruz Biotechnology	1:1000
10	Cyp1A1 (H00001543-D01P)	Novus Biologicals	1:100

### Confocal imaging

HT29 or CaCo2 or BMDM cells (50,000 cells/well) were plated on to 8-well chambered slides (154534PK; ThermoFisher Scientific) allowed them to grow overnight. The cells were induced with vehicle (0.01% DMSO) or UroA (50 µM) or UAS03 (50 µM) for desired time points and fixed with cold methonol. The AhR or Nrf2 or Cldn4 stained with respective anti-bodies (1:200 dilution) followed by fluorecently labeled (Alexa flour 594 for AhR and Alexa flour 488 for Nrf2 and Cldn4) secondary ab (1:500 dilution; ThermoFisher Scientific). The nucleus was stained with DAPI (Sigma Aldrich). The confocal images were captured using Nikon A1R confocal microscope using ×60 magnification lense with appropriate laser channels.

### AhR-reporter assay

AhR-reported assay was performed using AhR Reporter Assay system (Indigo Biosciences). The AhR Reporter cells (expressing luciferase under AhR promoter) as well as positive control MeBio (AhR ligand) compound were provided in the kit. The cells were treated with Vehicle or UroA or UAS03 or ellagic acid or MeBio for 6 h and luminoscence was measured according to manufacture’s instructions.

### Nrf2-reporter assay

ARE-luciferase plasmid vector was obtained from Cayman Chemicals. HT29 cells were transfected at 50% confluency using lipofectamine 3000 reagent (ThermoFisher Scientific). Briefly, cells were seeded in 6-well plates (0.5 × 10^6^ cells) and grown for 24 h. The transfection complex containing 1 µg of plasmid DNA and transfection reagent was added to each well in absence of FBS. After 6 h medium containing 10% FBS was added and cells were incubated for another 16–18 h. These cells were treated with vehicle (0.01% DMSO) or UroA (50 µM) or UAS03 (50 µM) or sulforaphane (10 µM) for 24 h. After incubation with inducers, cells were lysed and firefly luciferase activities (luminiscence) were measured with Luciferase Assay System (Promega) using mutiwell plate luminometer (BMG, LABTECH).

### Measurements of Cyp1A1 enzyme activty (ex vivo)

Mice were treated with Vehicle or UroA or UAS03, BNF, or FICZ daily for 1 week at indicated concentration either through oral or i.p. route. After 1 week, mice were euthanized and the colon and liver tissues were dissected. Microsomes from these tissues were prepared using the following procedure^66^. For hepatic microsomes, liver was first perfused with 0.9% sodium chloride solution and excised out. Adhering blood and saline was removed by blotting on tissue paper and tissue was homogenized in tissue homogenization buffer (50 mM Tris-HCl, pH 7.4 with 250 mM sucrose). Homogenate was centrifuged at 10,000 × *g* for 30 min at 4 °C. supernatant obtained was further centrifuged at 105,000 × *g* for 60 min at 4 °C. The pellet was washed with homogenization buffer and centrifuged again at 105,000 × *g* for 60 min at 4 °C. The pellet was suspended in homogenization buffer and used for protein and CYP assay. For intestinal microsome preparation, intestine was removed and washed with 0.9% sodium chloride. The intestine was longitudinally cut open to expose mucosal layer and mucosa was scrapped with help of glass slide. The scraped tissue was collected in homogenization buffer (50 mM Tris-HCl buffer containing glycerol (20% v/v), protease inhibitor (1%) and heparin (3 U/ml)). This suspended mucosa was homogenized and centrifuged at 10,000×*g* for 20 min at 4 °C. Supernatant obtained was further centrifuged at 105,000 × *g* for 60 min at 4 °C. The pellet was washed with buffer and centrifuged again at 105,000 × *g* for 60 min at 4 °C. The pellet was suspended homogenization buffer and used for protein and CYP enzymes assays.

### Ethoxyresorufin-O-deethylase (EROD) assay

The microsomal proteins (0.5 mg) were mixed with 200 μL Tris buffer (0.1 M, pH 7.4) containing ethoxyresorufin (0.01 mM). To start reaction, NADPH (0.1 mM) was added and incubated at 37 °C for 10 min. After 10 min, reaction was terminated by adding equal volume of acetonitrile and reaction mixture was centrifuged at 13,000 × g for 10 min at 4 °C. Supernatant was used to determine resorufin by measuring fluorescence (Ex. 530 nm, Em. 580 nm). Pure resorufin (Sigma Aldrich) was used to generate standard curve.

### P450-Glo Cyp1A1 luminiscence assay

The above microsomes (20 μg) were used for P450-Glo Cyp1A1 luminiscence assays as per manufacturer’s instructions.

### Measurement of Cyp1A1 enzyme activities in vitro

EROD assay: HT-29 cells (15,000 cells/well) treated with vehicle, UroA and UAS03 (24 h), were rinsed with HBSS buffer, and then fresh HBSS buffer was added along with 5 μM of 7-ethoxyresorufin. Cells were further incubated at 37 °C for 1 h. After the incubation time, fluorescence (Exc. 530 nm, Em. 580 nm) was measured and product (resorufin) formed was calculated from calibration standard and normalized with protein concentration.

P450-Glo Cyp1A1 luminiscence assay: HT29 cells (25,000 cells/well) were plated in 48 well plate. Cell were then treated with UroA (0.1, 1, 10, 25, and 50 µM) or UAS03 (0.1, 1, 10, 25, and 50 µM) or FICZ (0.1, 1, 10, 25, and 50 nM) for 24 h. After treatment, cells were washed to remove any residual drugs, and fresh medium containing Cyp1A1 substrate (as per protocol provided with kit Cat.# V8751; Promega) for 3 h. After incubation, 25 µl of culture medium was removed from each well and transferred to a 96-well white opaque plate and 25 µl of luciferin detection reagent was added to initiate the luminescence reaction and plate was incubated at room temperature for 20 min. After incubation, luminescence was recorded in luminometer. The data reported as fold change over vehicle treatment.

### Small interfering RNA (siRNA) mediated knockdown experiment

The AhR siRNA (SR300136) and Cyp1A1 siRNA (SR301093) was purchased from Origene. For knockdown experiments, HT29 cells (0.5 × 10^6^ cells/well) were plated in 6 well plate and grown for 24 h. The AhR, Cyp1A1 and control-siRNA was transfected into HT29 cells using Lipofectamine ® RNAiMAX reagent (ThermoFisher Scientific) as per intruction given. After 24 h of transfections, cell were induced with vehicle (0.01% DMSO), UroA (50 µM), and UAS03 (50 µM) for 24 h. After treatment with inducers, cells were lysed using RIPA buffer and total protein was used to analyse the expression of AhR, Cyp1A1 and Cldn4 by western blot.

### Cyp1A1 deletion by CRISPR/Cas9 method

HT29 cells (1.5 × 10^5^) were plated in 6-well in antibiotic free standard growth medium 24 h prior to transfection. At 60% confluency cells, cells were co-transfected with 2 µg each of CRISPR/Cas9 KO Plasmid (sc-400511-KO-2; Santa cruz) and HDR Plasmid (sc-400511-HDR-2; Santa cruz) using UltraCruz® Transfection Reagent (sc-395739; Santa Cruz). Medium was replaced with selective medium (containing 4 µg/mL puromycin) 96 h post transfection. Transfection was confirmed with fluorescence microscopy and western blot (CYP1A1). The double postive cells for GFP and RFP were sorted using MoFlo XDP sorting instrument (Beckman Coulter). The deletion of Cyp1A1 in these sorted was confirmed by western blots. These cells were then plated in 6-well plate for in standard medium for evaluating the effect of UroA/UAS03 on Cldn4 expression. After 24 h of UroA/UAS03 treatment cells were harvested for protein and Cldn4 expression was investigated along with normal HT29 cells.

### NF-κB EMSA assay

RAW 264.7 cells or BMDM were plated in 100 mm dishes (1 × 10^6^) in DMEM supplemented with 10% fetal bovine serum (FBS), 100 U/ml penicillin, and 100 U/ml streptomycin. Cells were allowed to grow for 24 h and after incubation, cells were treated with LPS (50 ng/mL) with and without UroA (50 µM) and UAS03 (50 µM) for 6 h. After treatments, culture medium was removed and washed with PBS. Cells were scraped and pelleted down in PBS. Supernatant was discarded and pellet was used for isolation of nuclear and cytosolic protein using NE-PER Nuclear and Cytoplasmic kit (Thermo Scientific; Cat #78833). Later nuclear protein (2 µg) was used for EMSA using Non-Radioactive EMSA Kits with IR Fluo-Probes for Nuclear factor kappa B p65 (Viagene Biotech Inc Cat # IRTF282 60).

### Colon explant culture

Colon tissue pieces (0.5–1 cm length) from wild type (C57BL/6) or Nrf2^−/−^ or AhR^−/−^ mice were cultured in triplicates for 24 h in complete DMEM-high glucose medium (supplemented with 10% fetal bovine serum, 1X penicillin-streptomycin solution) in a humidified atmosphere in the presence of vehicle (0.01% DMSO), UroA (50 µM) or UAS03 (50 µM). The tissues were processed for protein preparation (tissue lysates with RIPA plus buffer) or total RNA isolation. These tissue lysates or RNA were used to determine the expression of Nrf2, Cldn4 and AhR.

### Tissue processing for RNA and protein analysis

Mice were treated with as described in results section. Mice were euthanized with CO_2_ asphyxiation followed by cervical dislocation. Colon was dissected out and luminal contents were flushed out with cold PBS (containing PMSF and Sodium orthvandate). Small portion of colon was snap frozen in liquid nitrogen and stored at −80 °C for RNA analysis. For preparation of protein samples, colon was opened longitudinally and mucosa was scraped in ice-cold 1X PBS using pre-chilled glass slide and centrifuged at 300 × *g* for 10 min at 4 °C. Supernatant was discarded and pellet was suspended in RIPA buffer (containing 1X protease inhibitor) and vortexed at high speed. After 30 min incubation on ice, samples were centrifuged at 13,000 × *g* for 20 min at 4 °C. Supernatant was collected and protein was quantified using BCA protein quantification kit. The lysates were used appropriately for western blots.

### 28-day repeated dose toxicity study

To evaluate toxicity of UroA and UAS03, we performed 28-days repeated dose toxicity study. Mice were fed (oral gavage) with UroA (20 and 40 mg/kg/day) and UAS03 (20 and 40 mg/kg/day) daily for 28 days. Body weight, food, and water intake were assessed weekly. After 28 days, mice were killed and gross examination of all major organs were performed. Blood was collected to obtain serum. Serum alanine aminotransferase (ALT) and asparate aminotransferase (AST) were analyzed using ALT/ AST kit (BioVision) as per instructional manual.

### 2,4,6-Trinitrobenzenesulfonic acid (TNBS)-induced colitis

Male C57BL/6 or Nrf2^−/−^ mice (6–8 week old age mice) were anesthetized with ketamine/xylazine (100 mg/12.5 mg/kg IP) mixture and administered with single dose of TNBS (2.5 mg/mice; Sigma Aldrich, USA) in 50% ethanol. After administration of TNBS, mice were held upside down for 30–60 s to ensure proper distribution of TNBS in the colon. Control group received 50% ethanol without TNBS. Mice with TNBS were randomly divided into three groups, *viz*. vehicle (0.25% sodium carboxymethylcellulose (CMC)), UroA and UAS03. UroA or UAS03 was resuspended in 0.25% sodium-CMC at desired concentrations. The mice were given orally Veh or UroA or UAS03 in 100 μl at desired concentrations (4 or 20 mg/kg/body weight). The treatment started after 12 h of TNBS administration and every 12 h thereafter up to 72 h. The experiment was terminated post 60 h TNBS, where AhR^−/−^ mice were involved. In some experiments, we treated only once at post 12 h TNBS administration. TNBS administered and control mice were euthanized for tissue and plasma collection after 80 h of TNBS/ethanol treatment. Mice were examined for colitis phenotype.

### DSS-induced colitis

Acute experimental colitis in mice was induced by giving 3% (w/v) colitis grade DSS (MP Biomedicals) in drinking water for 7 days. Control animal received drinking water without DSS. All colitis group mice were randomly divided into three groups *viz*. vehicle treated (0.25% Na-CMC), UroA (20 mg/kg/day) and UAS03 (20 mg/kg/day) on the 4th and 6th day of DSS treatment. After 7 days, animals were put back on regular water for a period of 7 days. For chronic DSS colitis model, we used three cycles of 2.0% (w/v) DSS and each DSS cycle consisted or 7 days followed by 10 days of regular water and mice were treated with UroA (20 mg/kg/day) on every 4th and 6th day of DSS cycle.

### Assessment of colitis severity and tissue collection

Mice were evaluated daily for change in body weight, stool consistency, and rectal bleeding and score was given and combined to obtained disease activity index^67^. After euthanasia, the colon was removed and flushed with PBS containing (1 mM PMSF and 0.2 mM sodium orthovanadate). Colon length and colon weight were measured and small parts of colon were excised for myeloperoxidase (MPO) activity and RNA isolation. Tissues for MPO and RNA extraction were snap frozen in liquid nitrogen and stored in −80 °C until further analysis. Tissue for histological examination was stored in 10% phosphate buffered saline formalin. Blood was collected and serum was separated by centrifugation at 3500 × *g* for 15 min. Serum cytokines (IL-6, TNF-α; Biolegend) and chemokines (CXCL1; R&D Systems) levels were measured by ELISA according to manufacturer’s instructions.

### In vivo intestinal permeability assay

The gut barrier function was evaluated by in vivo intestinal permeability using FITC-Dextran (MW 4000; FD4, Sigma-Aldrich, USA)^68^. Briefly, mice were orally administered with FITC-dextran (60 mg/100 gm body weight). Mice were fasted for 4 h prior to euthanization. The FITC-dextran concentration in serum was determined using the standard curve of FITC-dextran in serum (excitation, 485 nm; emission, 525 nm; BMG LABTECH).

### Myeloperoxidase (MPO) activity

The MPO activity in the colons was determined using the following procedure^69^. Briefly, colon tissue was homogenized in 0.5% (w/v) hexadecyltrimethylammonium bromide (H6269; Sigma-aldrich, USA) in 50 mM PBS, pH 6.0. This homogenate underwent 3 freeze-thaw cycles and 10–15 s sonication to obtain homogenous suspension. The supernatant from this suspension was collected after centrifugation at 13000 × *g* for 20 min at 4 °C. The supernatant (10 µl) was then added to 50 mM potassium phosphate buffer (pH 6.0) containing 0.167 mg/ml *o*-dianisidine (Sigma-Aldrich, USA) and 0.0005% H_2_O_2_ (Sigma-Aldrich USA) and absorbance was taken at 450 nm (BMG, LABTECH) at 2 min interval. Units of MPO in each sample was determined by considering that one unit (U) of MPO = 1 µmol of H_2_O_2_ split with molar extinction coefficient of 1.13 × 10^−2^ nm/min and MPO in each sample calculated by using [ΔA(t_2_ − t_1_)]/Δmin × (1.13 × 10^−2^) formula and MPO units were normalized with per mg tissue.

### Histopathology

Collected colon tissue were fixed in 10% buffered formaldehyde solution overnight and fixed tissue underwent standard histopathological processing. Briefly, after fixation tissue underwent dehydration and cleaning with xylene before paraffin embedding. The paraffin section of 5 µm were cut (Leica microtome) and stained for H&E staining. The H&E images were captured using Aperio Scanscope. H&E sections were scored blindly using index scoring described by Erben et al.^70^.

## Supplementary information

Supplementary Information

Description of Additional Supplementary Files

Supplementary Data 1

Source Data

## Data Availability

The RNA-seq data described in this MS is deposited with GEO # GSE113581. The hyperlink for RNA seq as follows: https://urldefense.proofpoint.com/v2/url?u=https-3A__www.ncbi.nlm.nih.gov_geo_query_acc.cgi-3Facc-3DGSE113581&d=DwIBAg&c=OAG1LQNACBDguGvBeNj18Swhr9TMTjS-x4O_KuapPgY&r=gYAe1Rux-xOAaWAT6YX2N_noYeYJBx7FLRBVFljZPt0&m=aQROwFtABYMDtrIp4k7mNRkL77nfCBUH4EqXd2nQLK0&s=RH4q-x-nZSE8JU035mWKJs4mmznYY-E3Nvx9CkS_QKs&e=. The full raw data that support the findings of this study are available from the corresponding author upon reasonable request. The Source Data underlying all the bar graphs in the Figures are provided in the Source Data File.
