# Supplementary material for: Enhancement of the gut barrier integrity by a microbial metabolite through the Nrf2 pathway
Source: Nat Commun. 2019 Jan 9;10:89. doi: 10.1038/s41467-018-07859-7 (PMC6327034; doi:10.1038/s41467-018-07859-7)
Supplement: Supplementary file 1 — Supplementary Information [file 41467_2018_7859_MOESM1_ESM.pdf]

# Supplementary Information

**‘Enhancement of Gut Barrier Integrity by a Microbial Metabolite through Nrf2 Pathway’ by**

**Singh et al.**

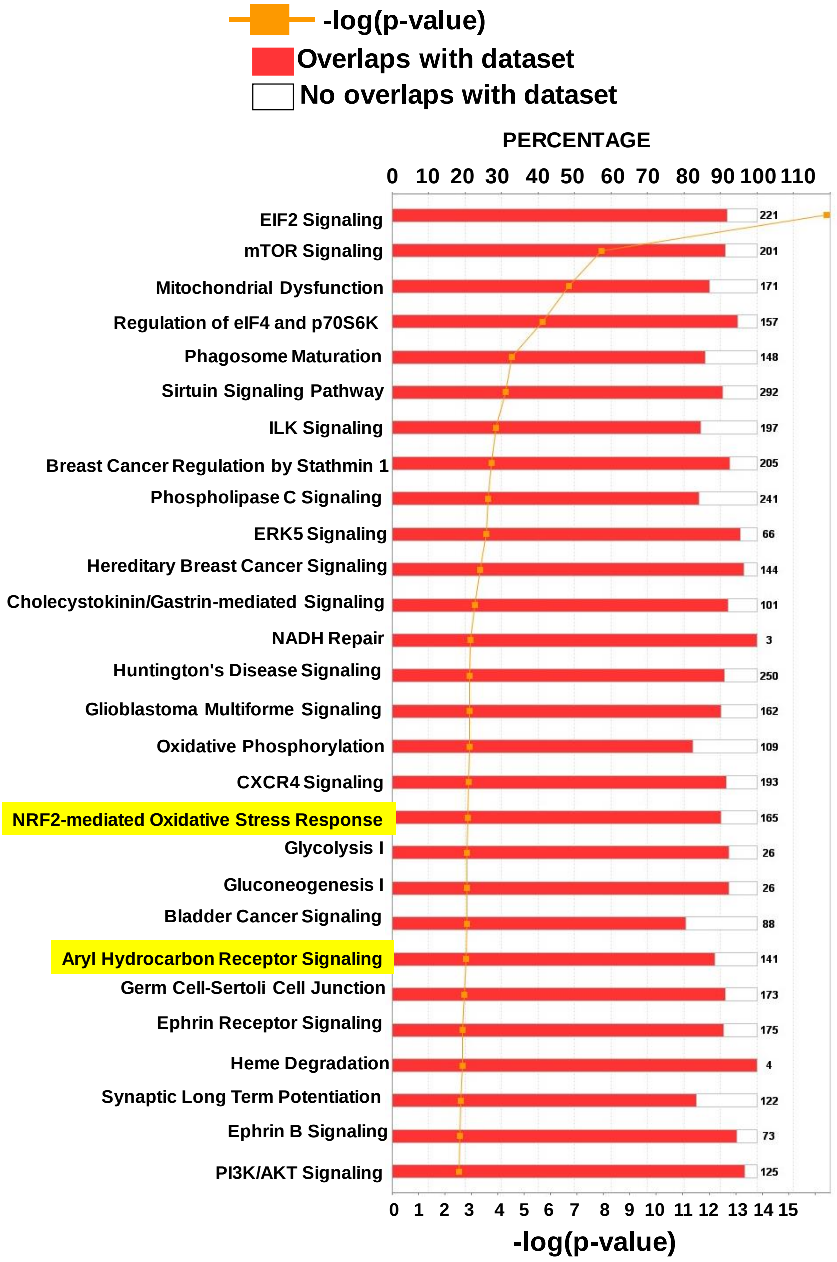


**Supplementary Figure 1. Canonical pathway analysis of RNA-Seq data.** HT29 cells were treated with Vehicle or UroA (50 µM) for 24 h and total RNA was isolated and performed RNA-Seq using Illumina HiSeq as described in methods. Pathway analysis was performed using Ingenuity pathway analysis by uploading genes that are significantly up or down regulated ($p<0.05$). Pathways that are more than 2.5 log p-value are shown. AhR and Nrf2 pathways that are of interest are highlighted.

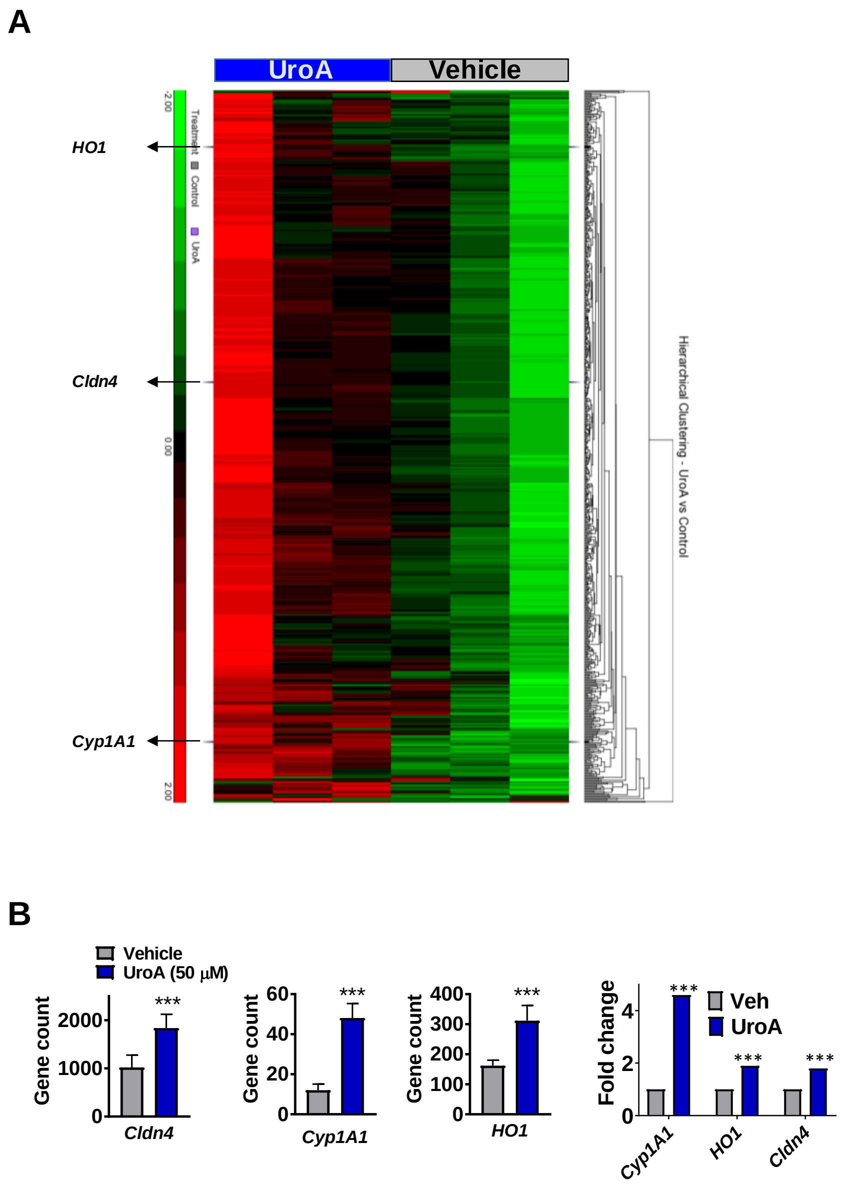


**Supplementary Figure 2. RNA-seq analysis-UroA upregulated *HMOX1, CLDN4* and *CYP1A1* genes.** HT29 cells were treated with Vehicle or UroA (50 μM) for 24 h and total RNA was isolated and performed RNA-Seq using Illumina Next Seq 500 as described in methods. **(A)** Genes with a LogFc > 0.8 were clustered using Euclidian Distance as dissimilarity measure. Genes of interest are highlighted from left: *HMOX1, CLDN4, CYP1A1*. Heat map generated using Partek program. **(B)** Gene counts and respective fold changes of selected genes, Claudin 4 (Cldn4), Cytochrome P450 1A1 (Cyp1A1) and hemoxygenase (HMOX1, HO1) are shown. Source data are provided as a Source Data File.

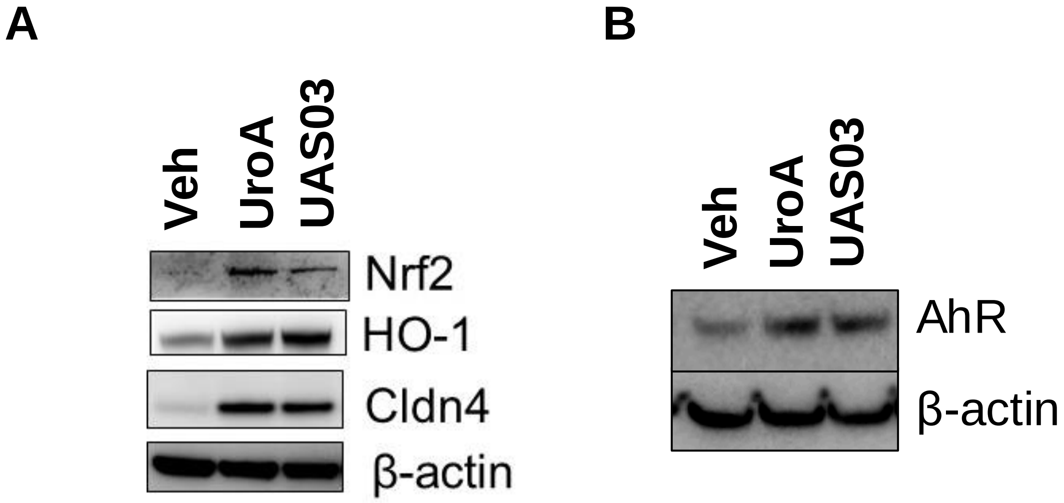


**Supplementary Figure 3. UroA/UAS03 treatment upregulate expression of Cldn4, HO1, Nrf2 and AhR in colons of mice.** C57BL/6 mice (7-8 week age old mice, n=3-4) were orally supplemented daily with Vehicle or UroA or UAS03 (20 mg/kg/bodyweight) for 7 days. The protein lysates were prepared from colon scrapes and subjected to immunoblots and detected the expression of the (A) Nrf2, HO1, Cldn4 and (B) AhR using appropriate antibodies as described in methods.

# 7-ethoxyresorufin-O-deethylase (EROD) assay

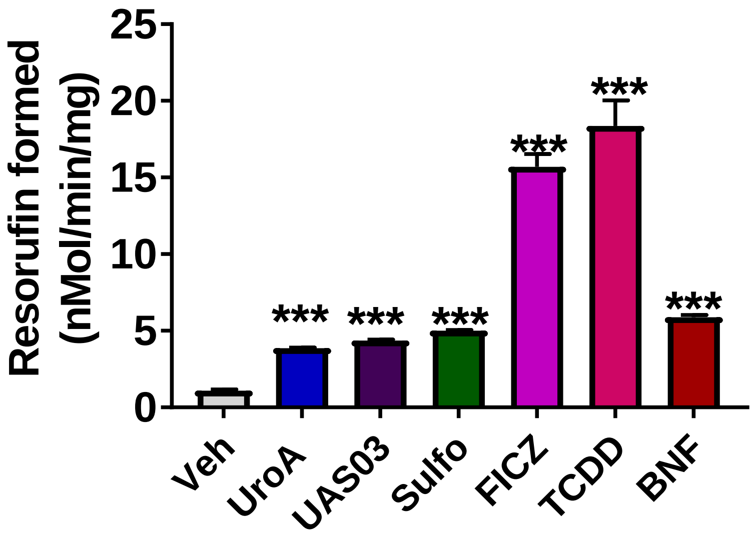


**Supplementary Figure 4.** HT29 cells treated (quadruplicate wells) with UroA or UAS03 (50 µM) or TCDD (10 nM) or FICZ (100 nM) or BNF (50 µM) for 24 h and Cyp1A1 enzyme activity was measured using EROD assay as described in methods. Statistics performed using unpaired t-test using Graphpad Prism software. Error bars, ±SEM; ***p<0.001; **p<0.01; **p<0.05. Source data are provided as a Source Data File.

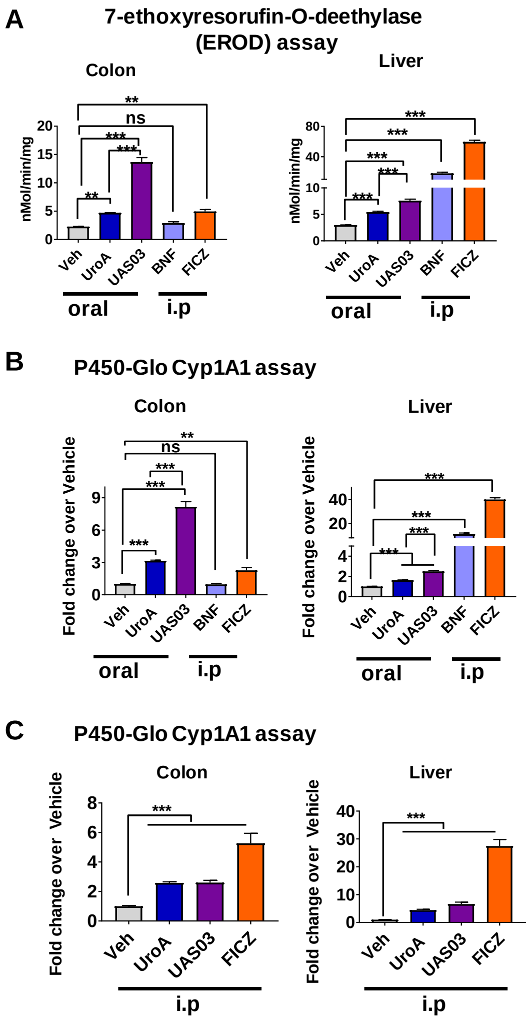


**Supplementary Figure 5. *In vivo* Cyp1A1 enzyme activities. (A-B).** C57BL/6 (n=3, 7 week old age) mice were treated orally with Vehicle (0.25% Na-CMC), or UroA (20 mg/kg/day) or UAS03 (20 mg/kg/day) for one week. β-naphthoflavone (BNF, 40 mg/kg/day) or 5,11-Dihydroindolo[3,2-*b*]carbazole-6-carboxaldehyde (FICZ, 1 μg/mouse/day) were delivered i.p daily for one week. At day 7, the mice were euthanized and microsomes from colon and liver were isolated. The Cyp1A1 enzyme activity was measured using EROD and P450-Glo Cyp1A1 assay methods. **C.** C57BL/6 (n=3, 7 week old age) mice were treated i.p. with Vehicle or UroA (5 mg/kg/day) or UAS03 (5 mg/kg/day) or FICZ, (1 μg/mouse/day) for week. The Cyp1A1 enzyme activities were measured from microsomes isolated from colon and liver tissues by P450-Glo Cyp1A1 assay method. Statistics were performed using unpaired t-test using Graphpad Prism software. Error bars, ±SEM *** $p<0.001$; ** $p<0.01$; *$p<0.05$. ns: not significant. Source data are provided as a Source Data File.

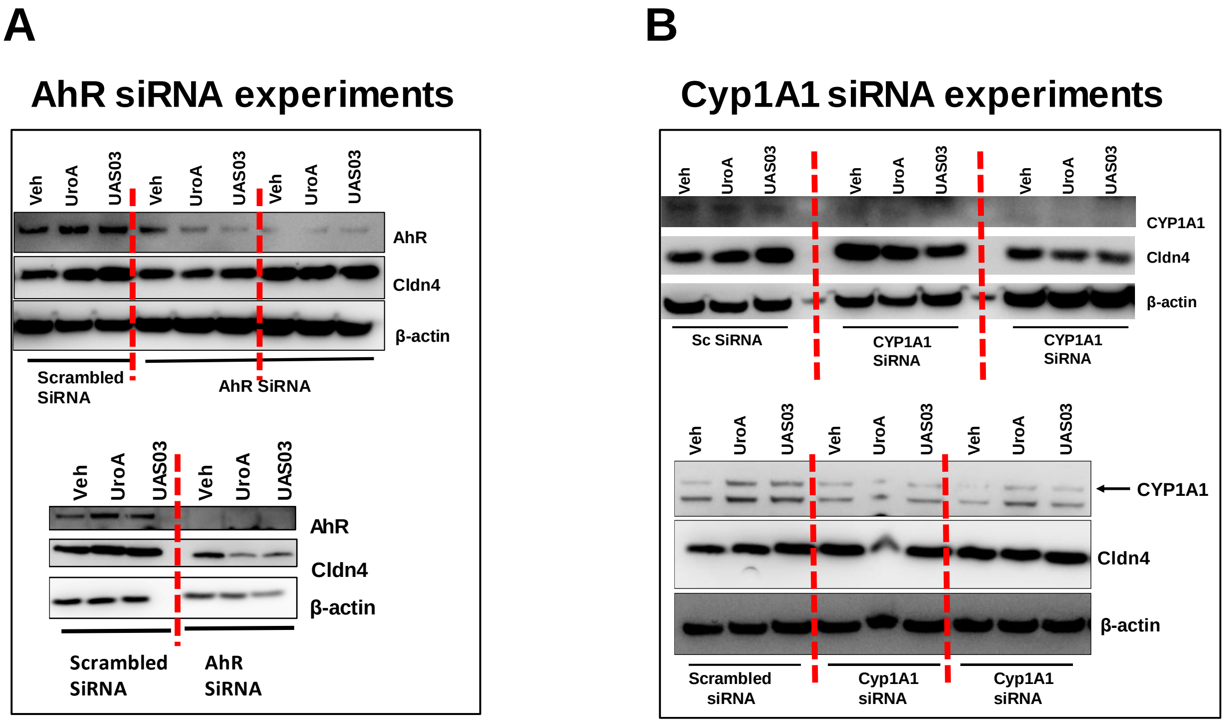


**Supplementary Figure 6. UroA/UAS03 induce Cldn4 in AhR and Cyp1A1 dependent manner.** Expression of **(A)** AhR or **(B)** Cyp1A1 was suppressed using respective siRNAs and Cldn4 expression was eavaluated upon UroA/UAS03 treatments as described in Figure 2H-I. These are additional biological replicates (n=3 for AhR siRNA and n=4 for Cyp1A1 siRNA) of immunoblots and were used for quantification in Figure 2H-I.

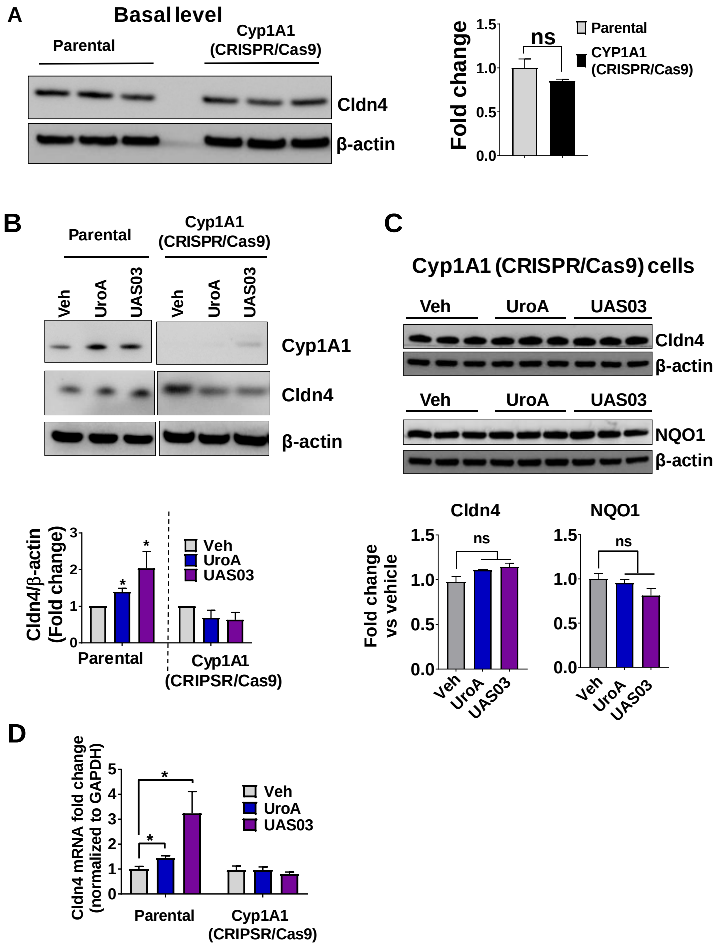


**Supplementary Figure 7. Expression of Cyp1A1 is required for UroA/UAS03 mediated upregulation of Cldn4.** Cyp1A1 gene was deleted using CRISPR/Cas9 method in HT29 cells. (**A**)**.** The basal level expression of Cldn4 were examined in parental HT29 or Cyp1A1 (CRISPR/Cas9) cells by Western blots and quantified. (**B**)**.** Parental HT29 or Cyp1A1 (CRISPR/Cas9) cells were treated with Veh or UroA or UAS03 (50 μM) for 24 h. Expression of Cyp1A1 and Cldn4 was measured by Western blots. Three independent replicates of Western blots were used to quantify band intensities by Image J software. **C.** Cyp1A1 (CRISPR/Cas9) cells (n=3) were treated with Veh or UroA or UAS03 (50 μM) for 24 h and examined expression of Cldn4 and NQO1 and quantified. The fold changes were calculated by setting up vehicle's Cldn4 or NQO1/β-actin ratio average as 1 and compared with treatments. **(D)** mRNA levels of Cldn4 were measured by Real time PCR from treatments as described in panel B. Statistics were performed using unpaired t-test using Graphpad Prism software.  Error bars, ±SEM *p<0.05; ns: not significant. Source data are provided as a Source Data File.

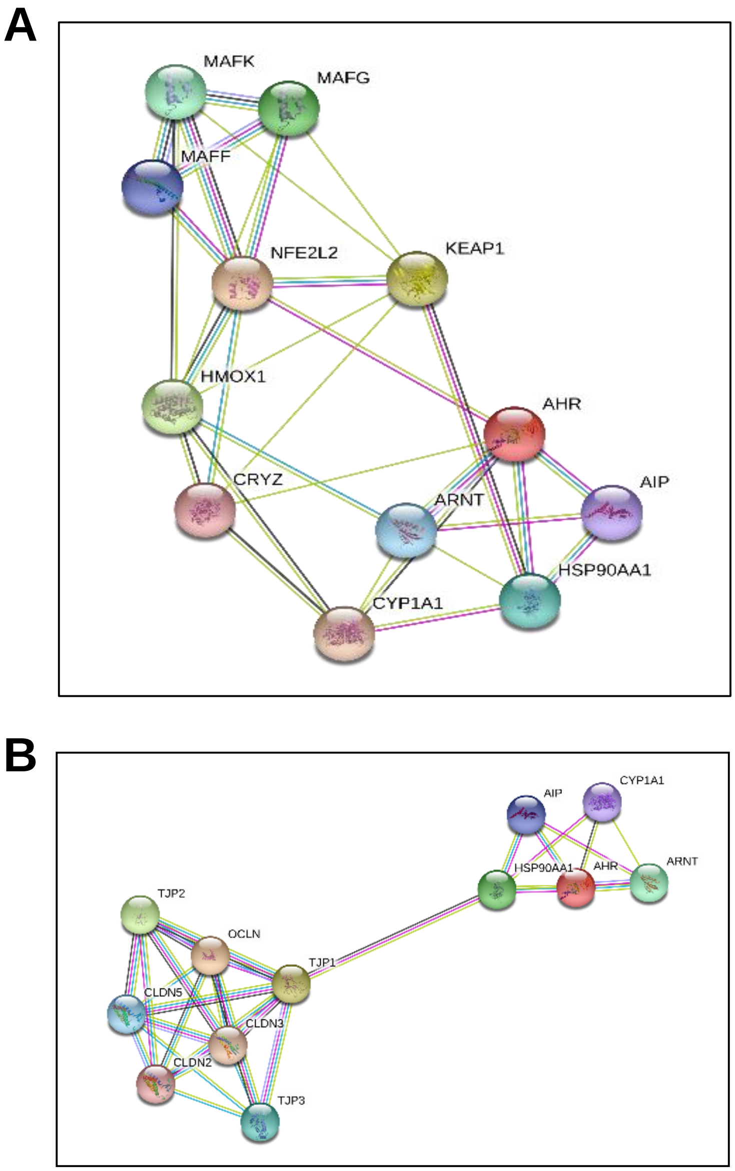


**Supplementary Figure 8. AhR-ligand Chip-seq analysis of MCF-7 cells**. Publically available ChIP-Atlas (http://chip-atlas.org/target_genes) Chip-seq analysis of AhR ligand. (**A)** AhR targets Nrf2 and Cyp1A1 genes. (http://dbarchive.biosciencedbc.jp/kyushu-u/hg19/target/AHR.1.html). (**B)** AhR also target tight junction proteins such as TJP1, 2, 3, Ocln, Clnd2,3 and 5.

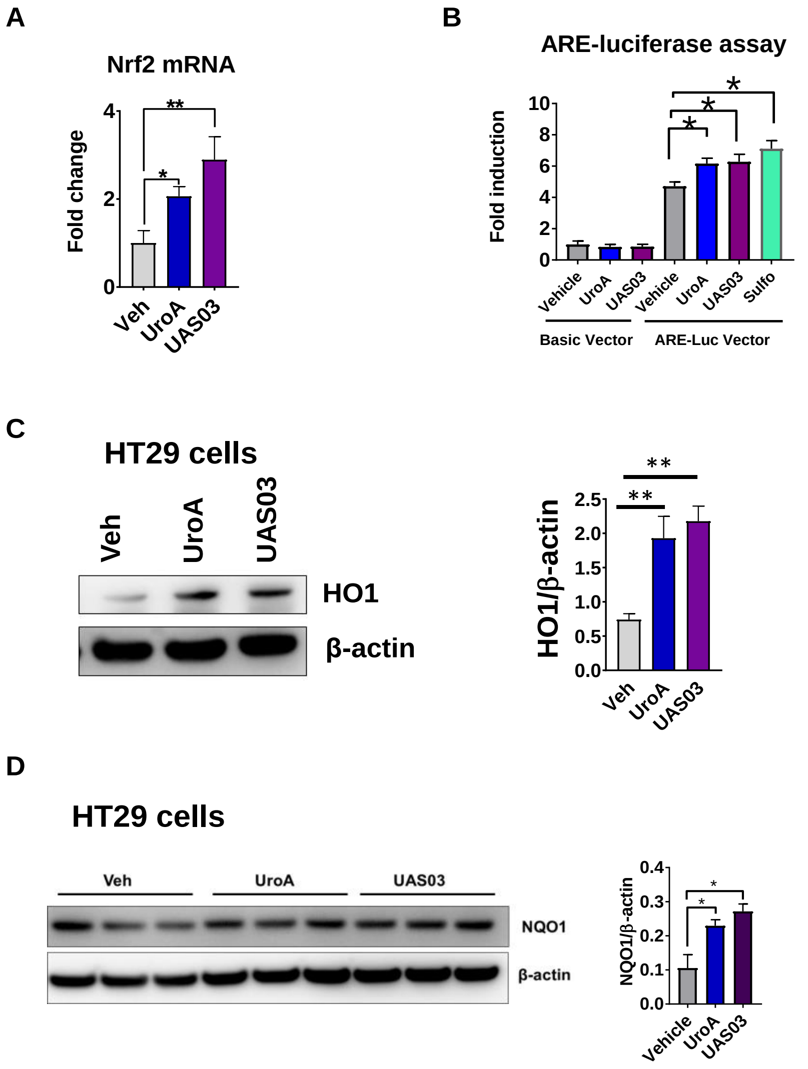


**Supplementary Figure 9. UroA and UAS03 upregulate expression of Nrf2-target genes. (A)** HT29 cells were treated with Vehicle or UroA or UAS03 (50 μM) (n=3) and total RNA was isolated. The mRNA levels of Nrf2 was evaluated using SyBR green real time PCR method. β-actin was used to normalize the expression. **(B)** HT29 cells were transfected with ARE-luciferase vector. Post 24 h transfection, cells were treated with Veh/UroA/UAS03 (50 μM) or sulforaphane (10 μM) for 24 h (n=3) and luminescence was measured. **(C-D)** HT-29 cell lysates prepared similar to described above and immunoblotted for HO1 using anti-HO1 (C) and NQO1 (D) using anti-NQO1. The quantification of immunoblots were performed using Image J software. Statistics performed using unpaired t-test using Graphpad Prism software. Error bars, ±SEM. **p<0.01; *p<0.05. Source data are provided as a Source Data File.

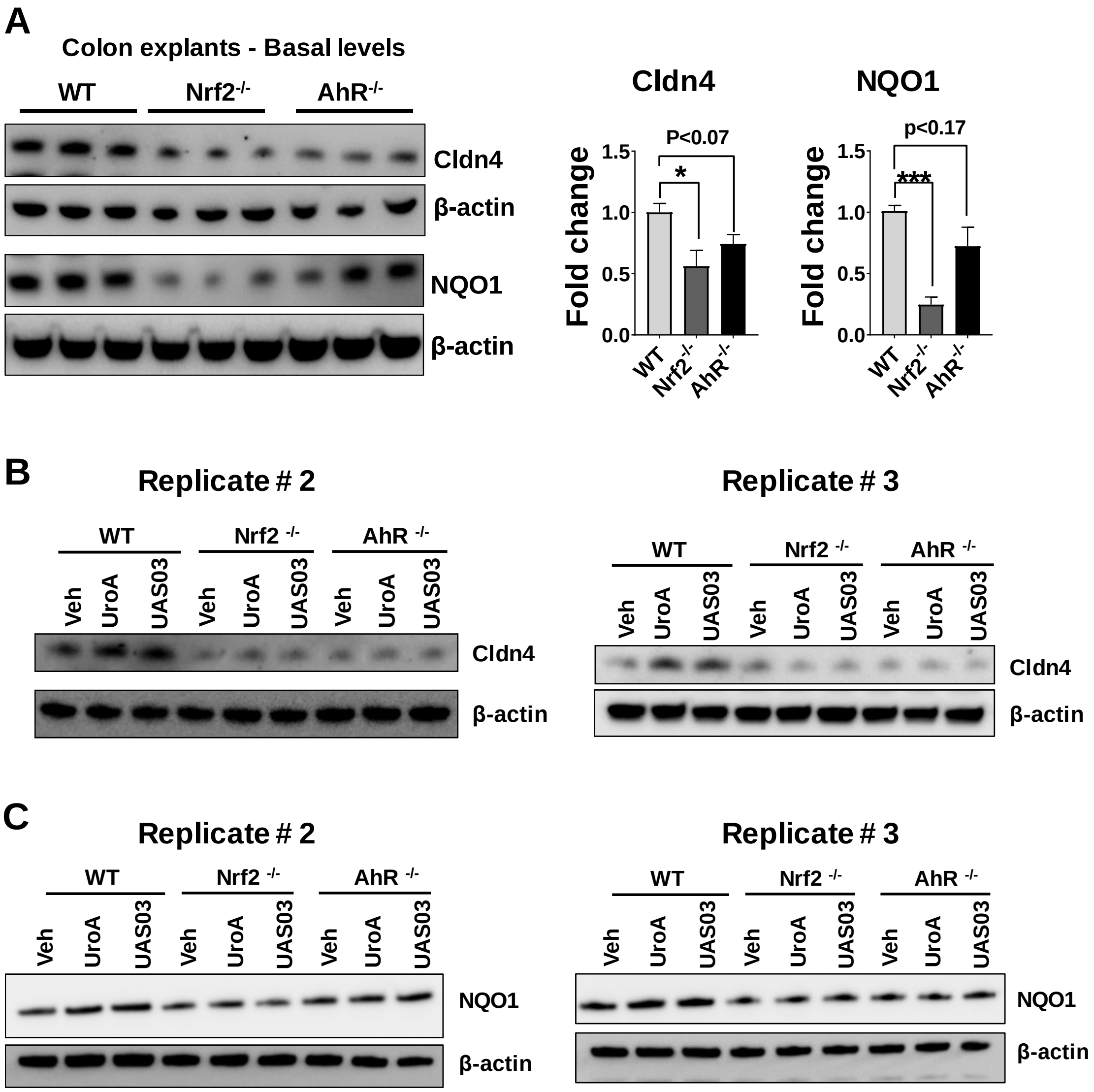


**Supplementary Figure 10. UroA/UAS03 induce Cldn4 in AhR and Nrf2 dependent manner (*Ex vivo* colon explants study).** These experiments represent biological replicates from different mice. The colon explants from WT and $AhR^{-/-}$ mice (A), and WT and $Nrf2^{-/-}$ mice (B) were prepared and induced with vehicle or UroA (50 µM) or UAS03 (50 µM) or FICZ (100 nM) for 24 h and measured the expression of indicated proteins. These are replicates supporting the data from Figure 3D. . Statistics performed using unpaired t-test using Graphpad Prism software. Error bars, ±SEM ***p<0.001; *p<0.05. Source data are provided as a Source Data File.

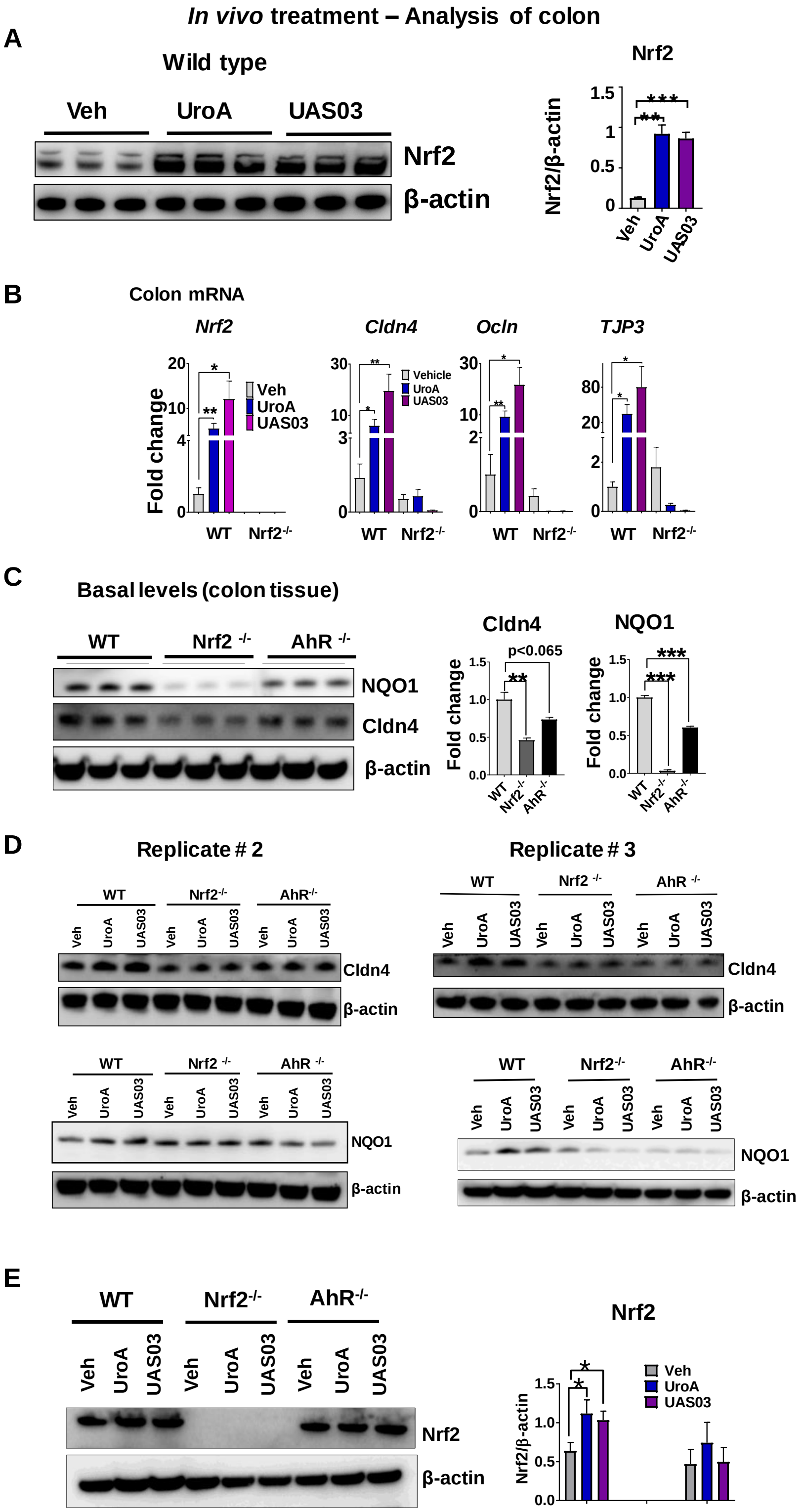


**Supplementary Figure 11. UroA/UAS03 induce Cldn4 in AhR and Nrf2 dependent manner (*In vivo* treatment studies).** C57BL/6 (WT), Nrf2$^{-/-}$ and AhR$^{-/-}$ mice (n=4-6) were treated with

vehicle (0.25% Na-CMC) or UroA (20 mg/kg/daily) or UAS03 (20 mg/kg/daily) for one week. Colons were isolated and scrapped the villi and the total protein was extracted as described in methods. **A.** Nrf2 is upregulated upon UroA/UAS03 treatment of wild type mice. **B.** Changes in mRNA levels of *Nrf2, Cldn4, Ocln and TJP3* in colon tissues (WT and Nrf2$^{-/-}$ mice) were measured using RT PCR. **C.** Colons from untreated C57BL/6 (WT), Nrf2$^{-/-}$ and AhR$^{-/-}$ mice (n=3) were isolated and the basal level of expressions of NQO1 and Cldn4 were measured by immunoblots as described in methods. **D.** C57BL/6 (WT), Nrf2$^{-/-}$ and AhR$^{-/-}$ mice (n=4) treated with vehicle (0.25% Na-CMC) or UroA (20 mg/kg/daily) or UAS03 (20 mg/kg/daily) for one week. Colon tissue lysates were prepared and examined expression of Cldn4 and NQO1 by Western blots. UroA/UAS03 failed to upregulate Cldn4 and NQO1 in Nrf2$^{-/-}$, AhR$^{-/-}$ mice. The immunoblots represent independent replicates to support the data in Figure 3F. **E.** Expression of Nrf2 was determined in same samples described in panel D by immunoblots. Immunoblots (n=3) were quantified using Image J and represented ratio of Nrf2/β-actin. . Statistics performed using unpaired t-test using Graphpad Prism software. Error bars, ±SEM ***p<0.001; **p<0.01; *p<0.05. Source data are provided as a Source Data File.

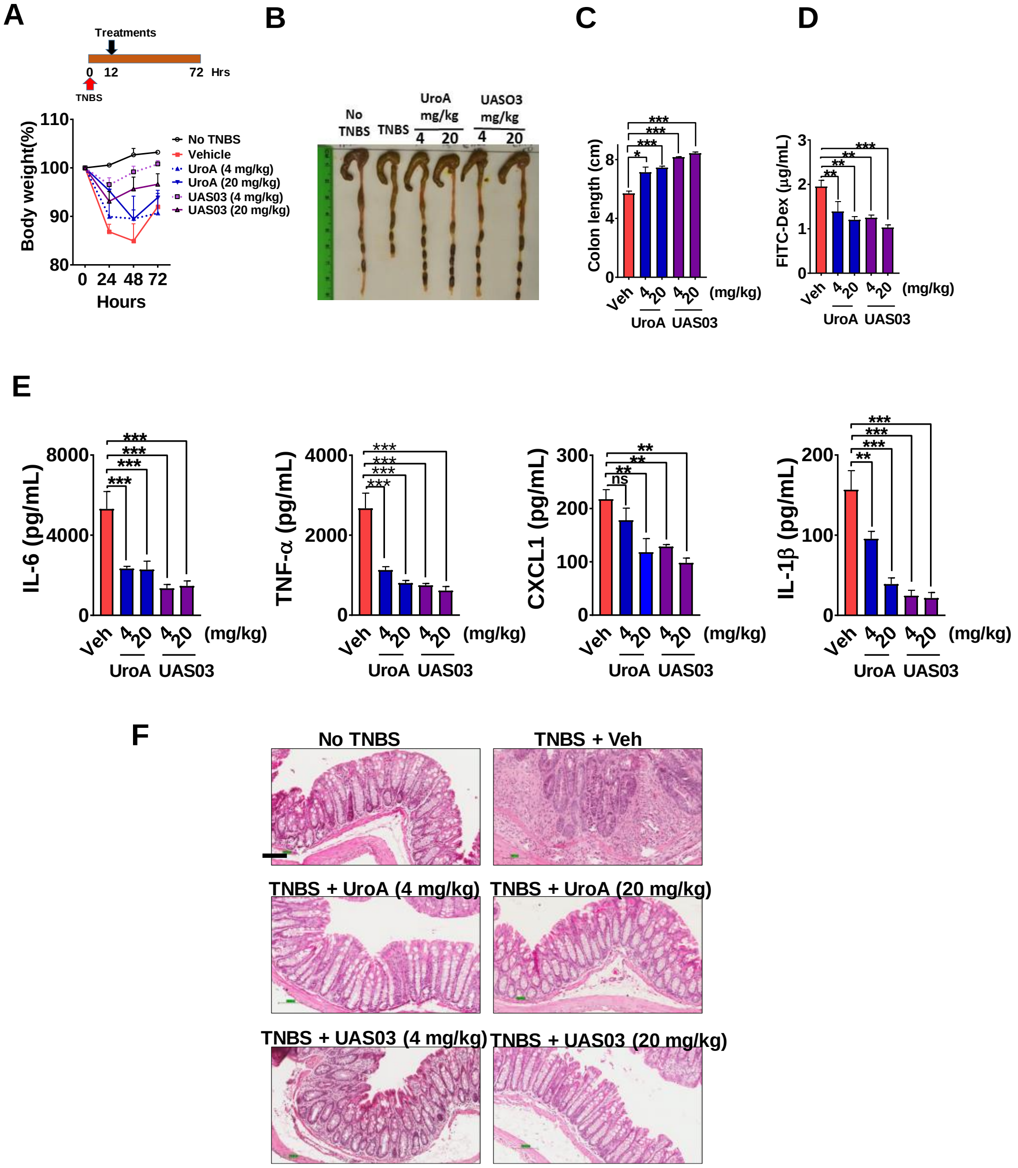


**Supplementary Figure 12. Single dose of UroA and UAS03 protects against TNBS-induced colitis in mice.** TNBS (2.5 mg/mouse) was administered intrarectally in C57BL/6 male (n=3-4/ group) to induce colitis. A single dose of UroA (4 or 20 mg/kg bodyweight) or UAS03 (4 or 20 mg/kg body weight) or vehicle (0.25% sodium carboxymethylcellulose) were administered orally post 12 h of TNBS instillation. Mice were euthanized at 72 h post TNBS administration. **(A)** Experimental design and changes in body weights (%) are shown. (No TNBS- Solid black line; Veh+TNBS- Solid red line; UroA (20 mg/kg) + TNBS- Solid blue line; UAS03 (20 mg/kg) +TNBS- Solid purple line; UroA (4 mg/kg) + TNBS- dashed blue line; UAS03 (20 mg/kg) +TNBS- dashed purple line). **(B)** Representative colon images **(C)** colon lengths are shown. **(D)**

Intestinal permeability was measured using FITC-dextran permeability assay. **(E)** Serum cytokines IL-6, TNF-α, CXCL1 and IL-1β were measured using standard ELISA methods. **(F)** Representative H&E section images captured using Aperio Imagescope. Scale bar indicates 100 microns. Statistics performed using unpaired t-test using Graphpad Prism software. Error bars, ±SEM ***p<0.001; **p<0.01; *p<0.05. Source data are provided as a Source Data File.

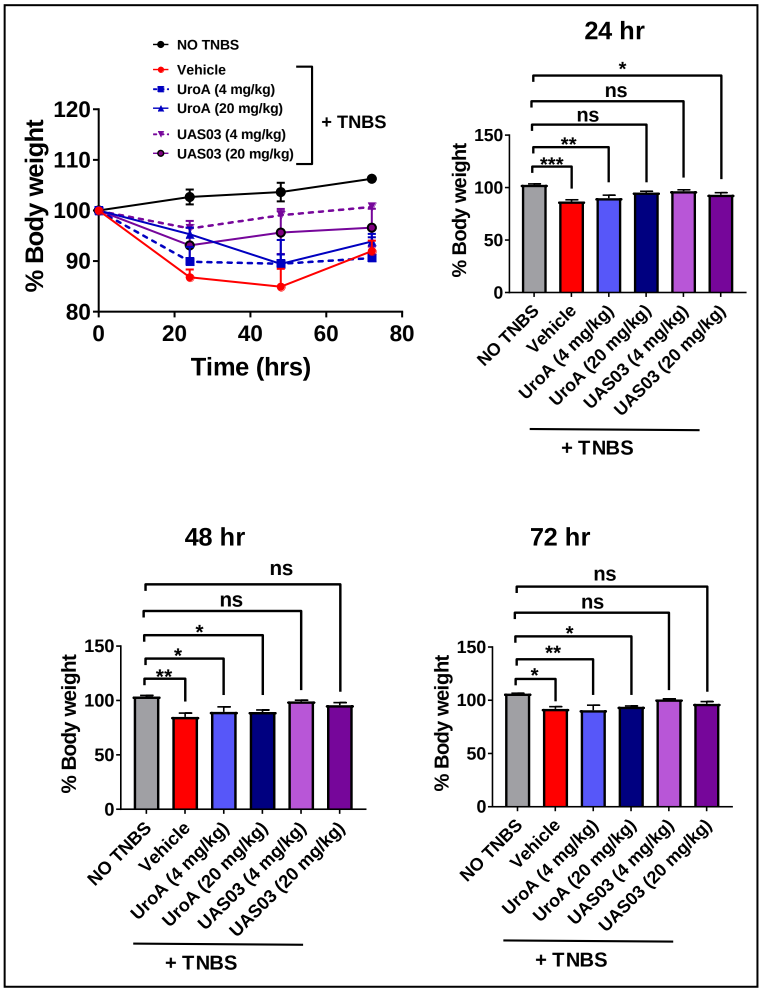


**Supplementary Figure 13. Detail analysis of percent body weight loss of data presented in Supplementary Figure 12A.** (No TNBS- Solid black line; Veh+TNBS- Solid red line; Pre-TNBS+UroA- Solid blue line; Pre-TNBS+UAS03- solid purple line; Post-TNBS+UroA- dashed blue line; Post-TNBS+UAS03- dashed purple line). The data is separately presented for each time point. Statistics performed using unpaired t-test using Graphpad Prism software. Error bars, ±SEM ***$p<0.001$; **$p<0.01$; *$p<0.05$. Source data are provided as a Source Data File.

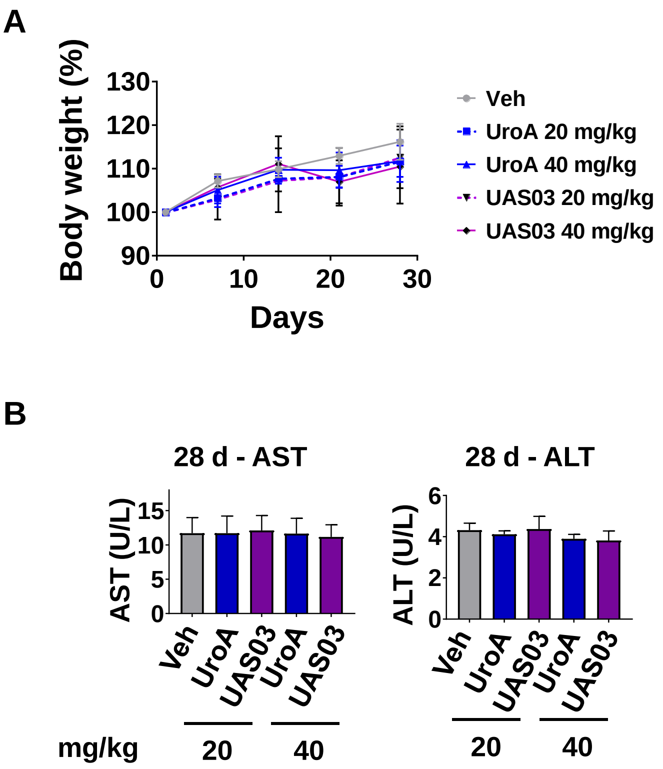


**Supplementary Figure 14. Evaluation of UroA/UAS03 toxicity in mice.**

C57BL/6 mice (7-8 week age old mice, n=4-5) were orally supplemented daily with Vehicle (Veh) or UroA or UAS03 (20 or 40 mg/kg/bodyweight) for 28 days. **(A)** Changes in body weight were measured once a week. (Vehicle- Solid gray line; UroA (20 mg/kg)- dashed blue line; UroA (40 mg/kg)- Solid blue line; UAS03 (20 mg/kg)- dashed purple line; UAS03 (40 mg/kg) – Solid purple line) **(B)** Serum AST and ALT levels at day 28 were measured using standard ELISA kits. Statistics performed using unpaired t-test using Graphpad Prism software and no significant differences were observed. Source data are provided as a Source Data File.

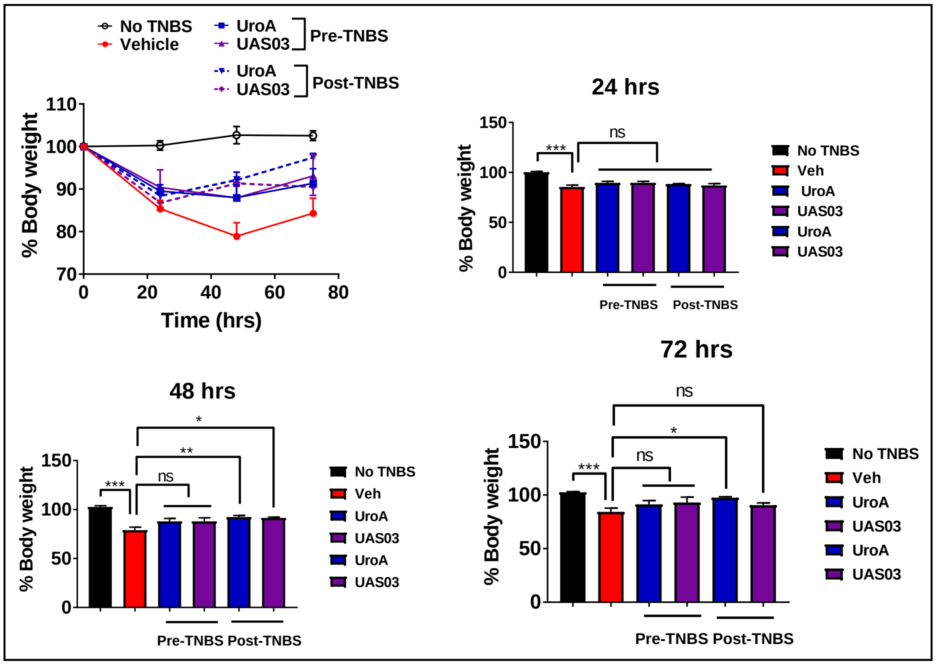


**Supplementary Figure 15. Detail analysis of percent body weight loss of data presented in Figure 5C.** The data is separately presented for each time point. . (No TNBS- Solid black line; Veh+TNBS- Solid red line; Pre-TNBS+UroA- Solid blue line; Pre-TNBS+UAS03- solid purple line; Post-TNBS+UroA- dashed blue line; Post-TNBS+UAS03- dashed purple line). Statistics performed using unpaired t-test using Graphpad Prism software. Error bars, ±SEM ***$p<0.001$; **$p<0.01$; *$p<0.05$. ns: not significant. Source data are provided as a Source Data File.

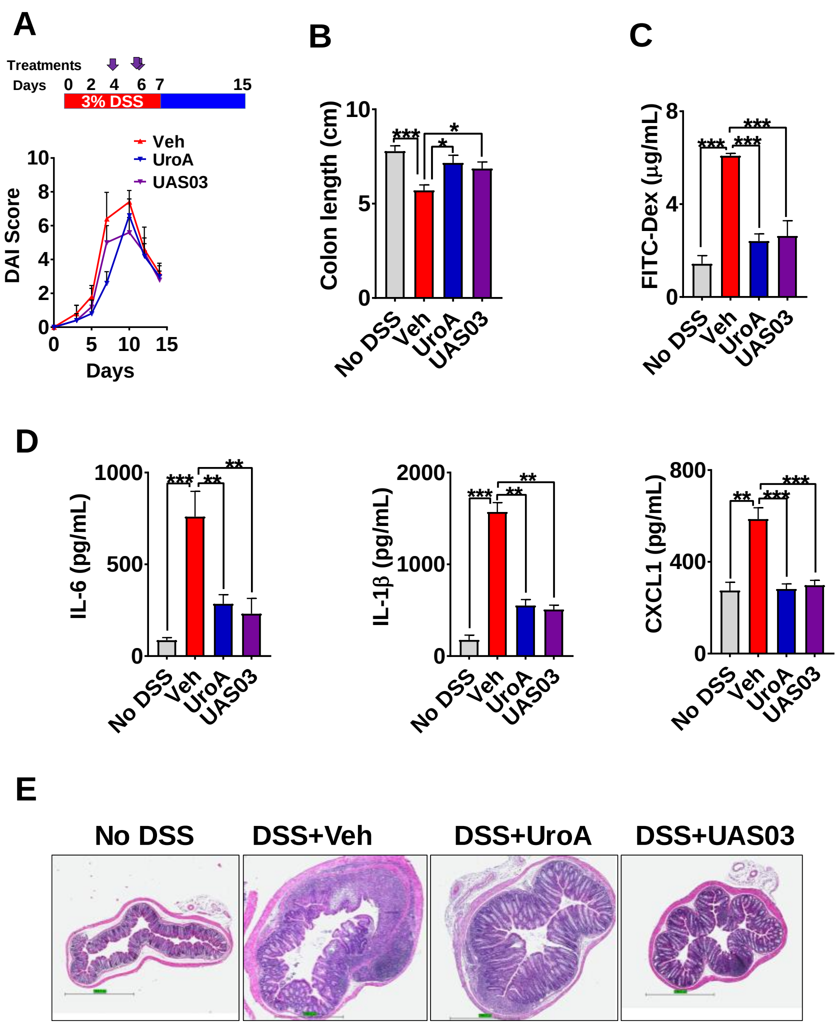


**Supplementary Figure 16. Treatment with UroA/UAS03 mitigates DSS-induced acute colitis.** C57BL/6 mice (7-8 week age old mice, n=8) were administered with 3% DSS in drinking water for 7 days and allowed them to recover for 7 days with normal water. UroA (20 mg/kg) or UAS03 (20 mg/kg) or vehicle (0.25% sodium carboxymethylcellulose) was delivered orally in 100 µl of volume on day 4 and 6 post-DSS. On 15$^{th}$ day, mice were euthanized and analyzed the colonic inflammation. **(A)** Experimental design and disease activity index (DAI) scores are shown (Vehicle- Solid red line; UroA- Solid blue line; UAS03-Solid purple line). **(C)** colon lengths, **(D)** Intestinal permeability with FITC-dextran assay were determined. **(E)** Serum cytokines were measured using standard ELISA kits. **(F)** Representative colon H&E section photomicrographs were captured using Aperio Image Scope. The scale bar indicates 1 mm. Statistics performed using unpaired t-test using Graphpad Prism software. Error bars, ±SEM ***$p<0.001$; **$p<0.01$; *$p<0.05$. Source data are provided as a Source Data File.

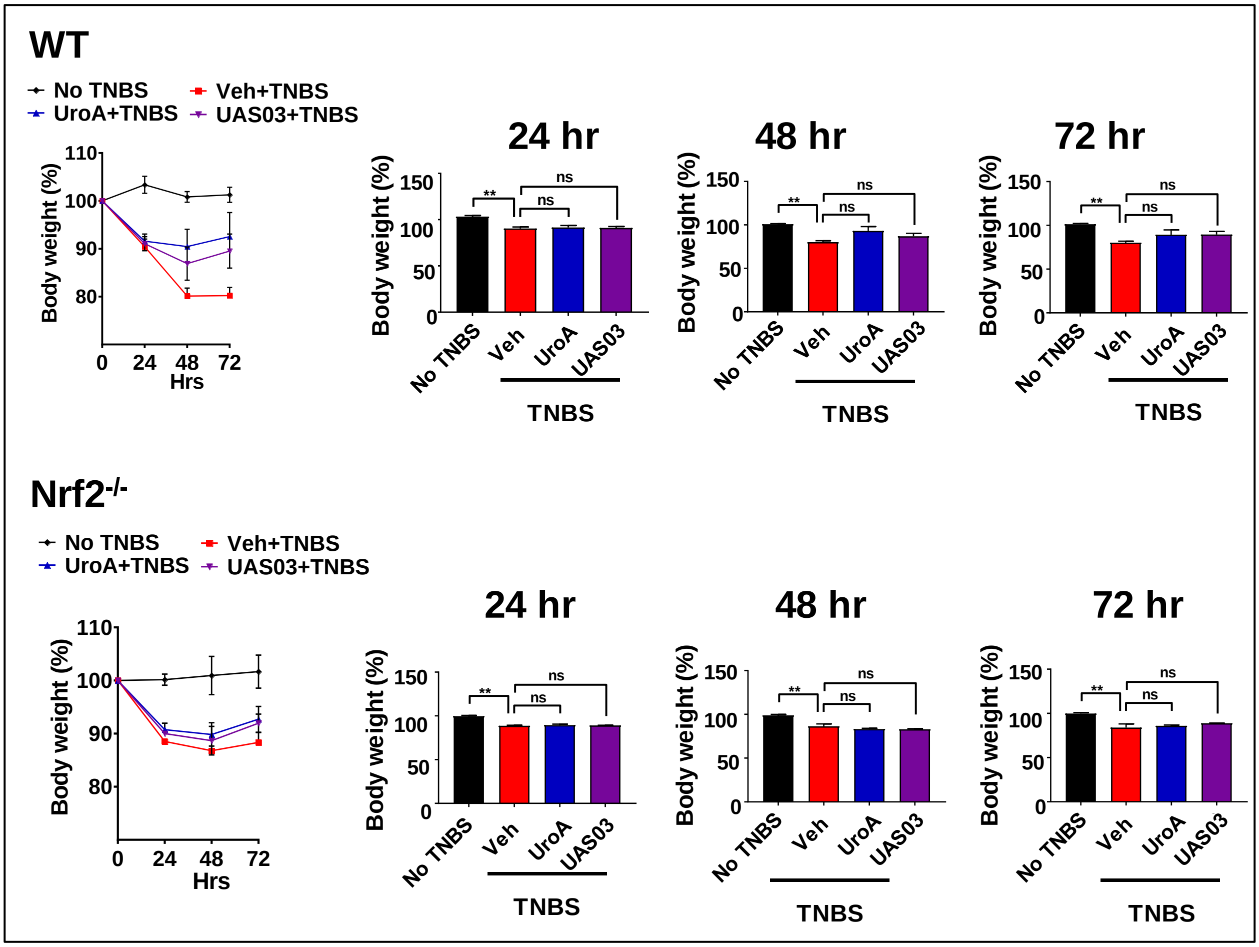


**Supplementary Figure 17. Detail analysis of percent body weight loss of data presented in Figure 7B.** The data is separately presented for each time point (No TNBS- Solid black line; Veh+TNBS- Solid red line; UroA+TNBS- Solid blue line; UAS03+TNBS- Solid purple line). Statistics performed using unpaired t-test using Graphpad Prism software. Error bars, ±SEM **p<0.01; ns: not significant. Source data are provided as a Source Data File.

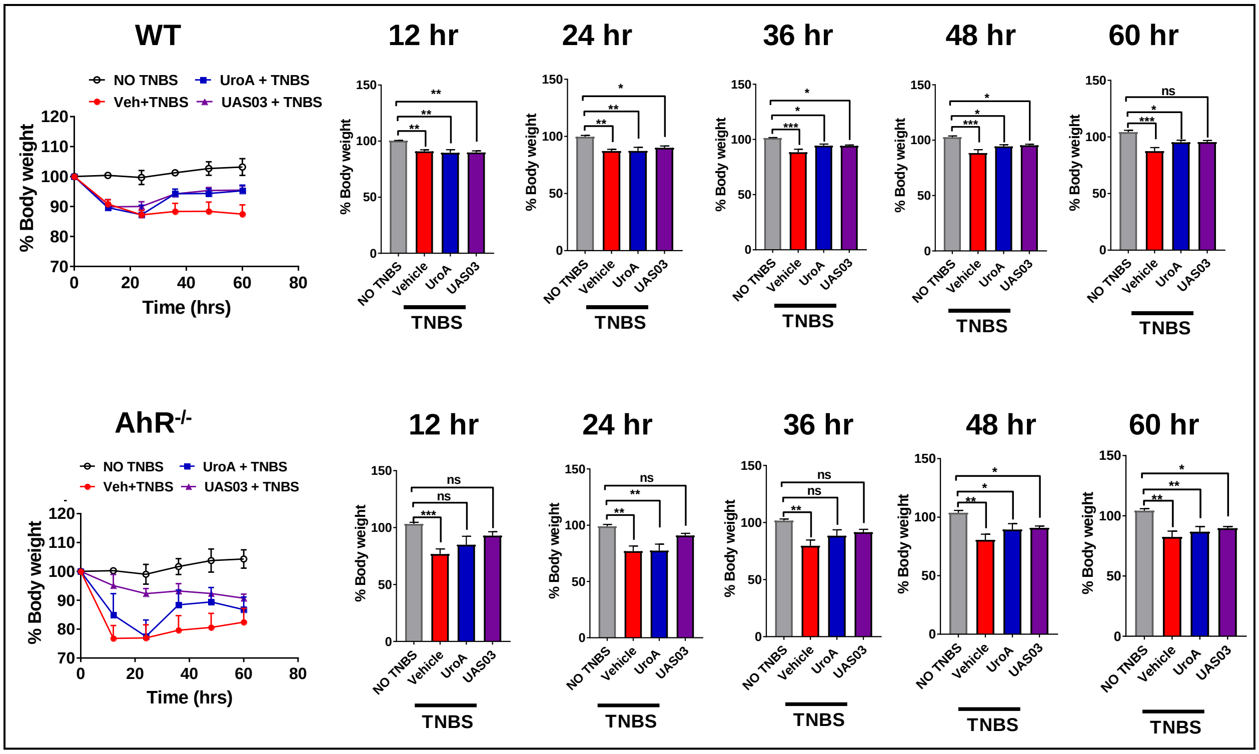


**Supplementary Figure 18. Detail analysis of percent body weight loss of data presented in Figure 8B.** The data is separately presented for each time point. (No TNBS- Solid black line; Veh+TNBS- Solid red line; UroA+TNBS- Solid blue line; UAS03+TNBS- Solid purple line). Statistics performed using unpaired t-test using Graphpad Prism software. Error bars, ±SEM ***p<0.001; **p<0.01; *p<0.05. Source data are provided as a Source Data File.

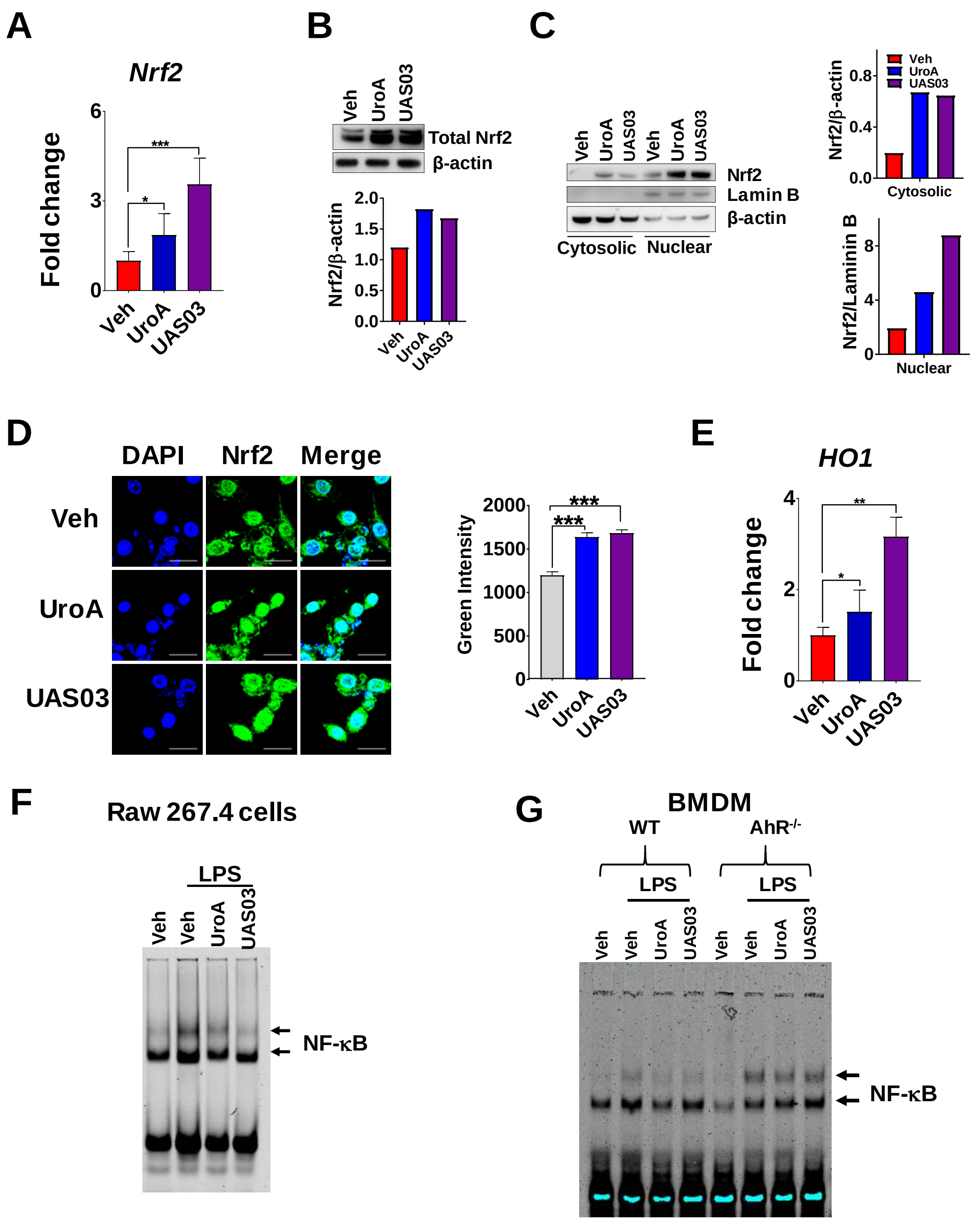


**Supplementary Figure 19. A-E. UroA/UAS03 activate Nrf2-dependent pathways in BMDM. (A)** Wild type BMDM were isolated and cultured for 7 days. BMDM were treated with Veh (0.01% DMSO) or UroA or UAS03 (50 μM) for 6 h and total RNA was isolated. Nrf2 levels were measured using SyBR green Real Time PCR method. **(B)** Veh or UroA or UAS03 treated BMDM cell lysates were immunoblotted for Nrf2. **(C)** The nuclear and cytosolic fractions of BMDMs were immnunobloted for Nr2. Lamin B and β-actin were used as normalizing proteins. **(D)** BMDM cells were grown on cover slip bottom fluorodishes overnight and treated with Vehicle or UroA

(50 μM) or UAS03 (50 μM) for 6 h. The expression of Nrf2 was determined by immunofluorescence staining using anti-Nrf2 followed by secondary antibody tagged with Alexa-488 dye and DAPI was used to stain nucleus. The fluorescence images were captured using Nikon A1R confocal microscope at 60x magnification and green fluorescence (n= >15 cells) was measured using Nikon elements software. The scale bar indicates 25 μm. Statistics performed using unpaired t-test using Graphpad Prism software. Error bars, ±SEM ***p<0.001; **p<0.01; *p<0.05 **(E)** BMDMs treated with Vehicle or UroA (50 μM) or UAS03 (50 μM) for 24 h and mRNA levels was estimated using SyBR RT PCR as described in methods. **F-G. UroA/UAS03 reduce LPS-induced NF-κB activation in AhR-dependent manner.** NF-κB activation was evaluated by EMSA assays. Raw 267.4 cells (F) or BMDMs were treated with LPS (50 ng/mL) in the presence or absence of UroA or UAS03 (25 μM) for 6 hr and nuclear extract (2 μg) was used to determine NF-κB binding by EMSA. Source data are provided as a Source Data File.

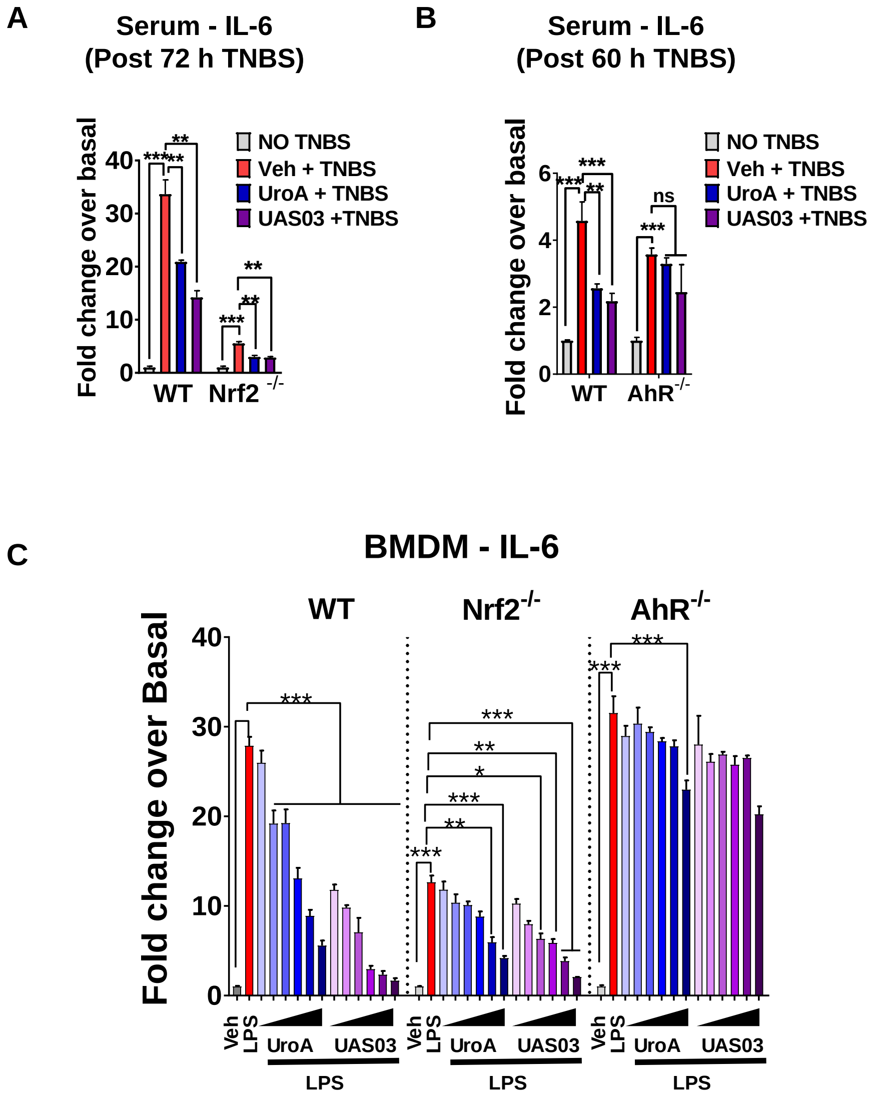


**Supplementary Figure 20. UroA/UAS03 mediate anti-inflammatory activities through AhR. A-B.** The IL-6 data represented as fold over its own basal level. The absolute values are provided in main figures (Fig. 7F and 8F). Serum IL-6 changes in TNBS-induced colitis model in (**A**) WT and Nrf2$^{-/-}$ mice; **(B)** WT and AhR$^{-/-}$ mice. (**C**). LPS-induced levels of IL-6 in the presence or absence of UroA/UAS03. The absolute values and detailed legend are provided in Figure 8H. The fold change over basal was calculated using its own control. WT+Veh or Nrf2$^{-/-}$+Veh or AhR2$^{-/-}$ +Veh were used to normalize to 1. Statistics performed using unpaired t-test using Graphpad Prism software. Error bars, ±SEM ***p<0.001; **p<0.01; *p<0.05. Source data are provided as a Source Data File.

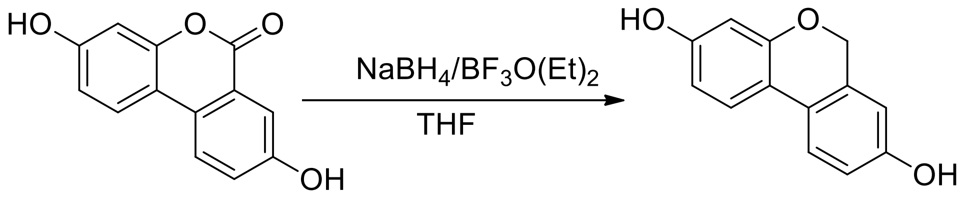


**Supplementary Figure 21. Synthesis of UAS03.** The compound UAS03 has been synthesized by reducing the lactone to cyclic ether as described in Methods section in the manuscript.

**Supplementary Figure 22:**

**Original full Western blots:**

All the antibodies were tested against cell lysates (Caco2 cells) and mouse colon tissue extracts. 25 μg of the protein was loaded in to the wells. The full gel – Western blots are shown below. In some of the experiments, the transferred membranes were cut according to their molecular weights and probed with indicated respective antibodies. Therefore, the β-actin (which was used as control) from the same gel are duplicated in couple of figures for ease of presentation purposes.

The following figures are from the same gel and have same β-actin as control.

1. Figure 1E – Cldn4 (23 kDa); Figure 3A – Nrf2 (~110 kDa)
2. Figure 1E – ZO1 (220 kDa); Figure S9D – NQO1 (31 kDa)

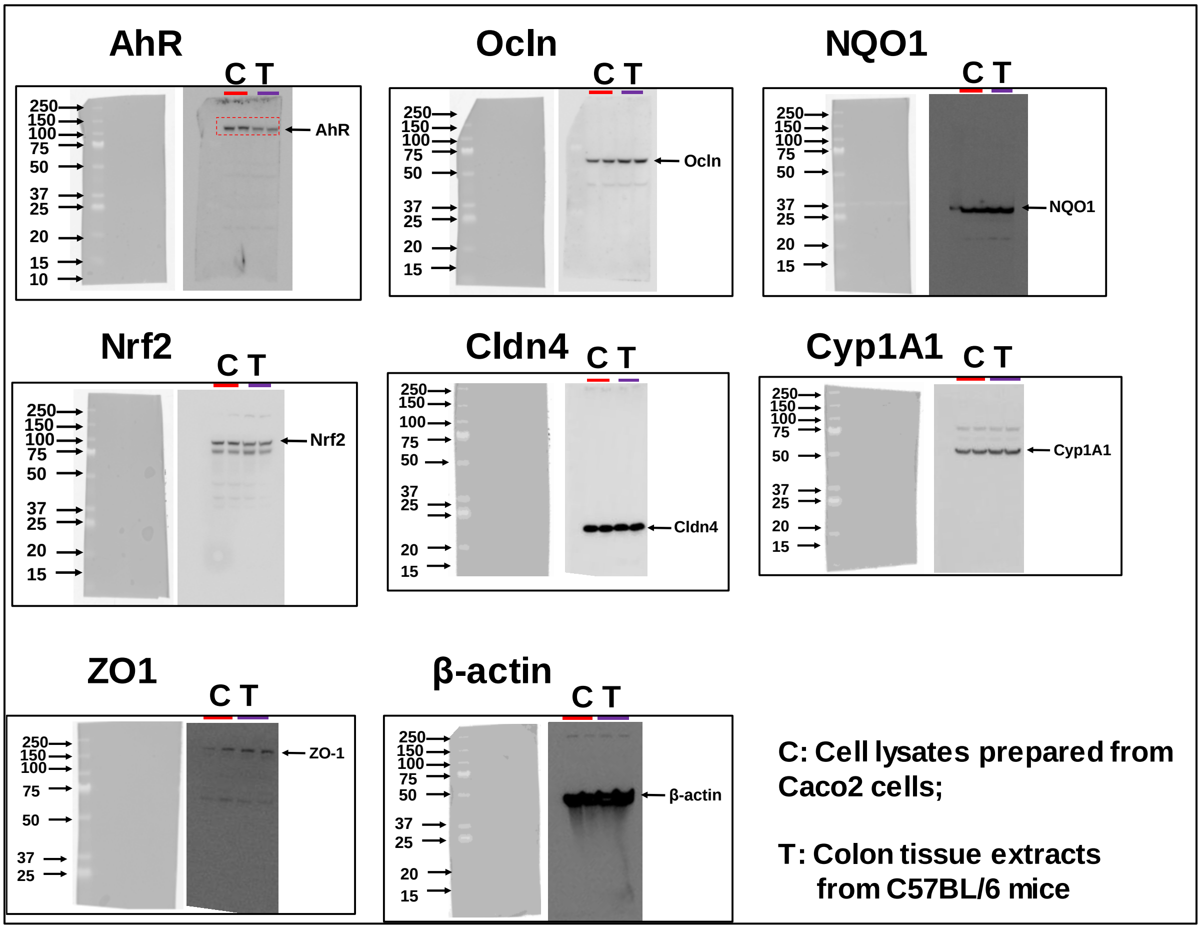


(Continued..)

## Figure 1E

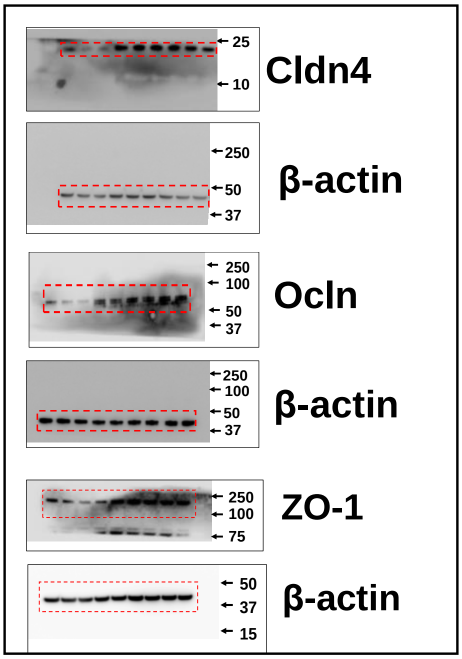


## Figure 2B

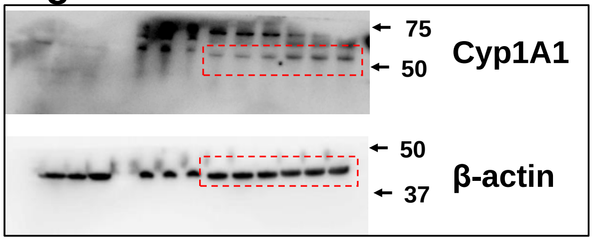


## Figure 2G

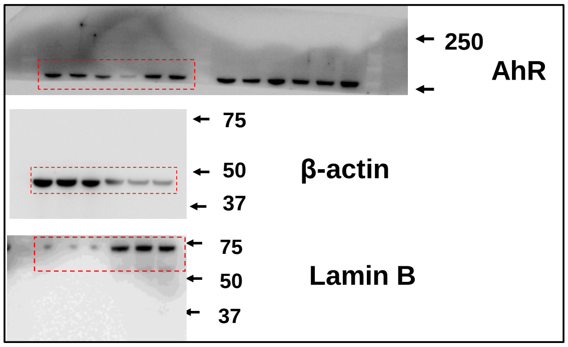


## Figure 2H

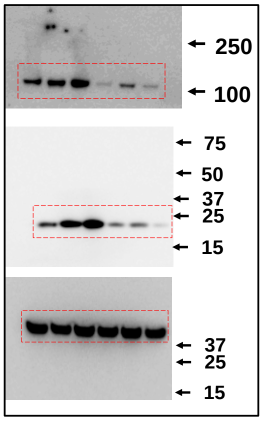


## Figure 3A

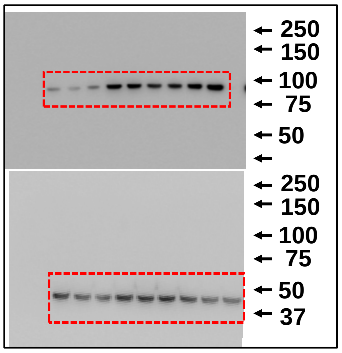


## Figure 3B

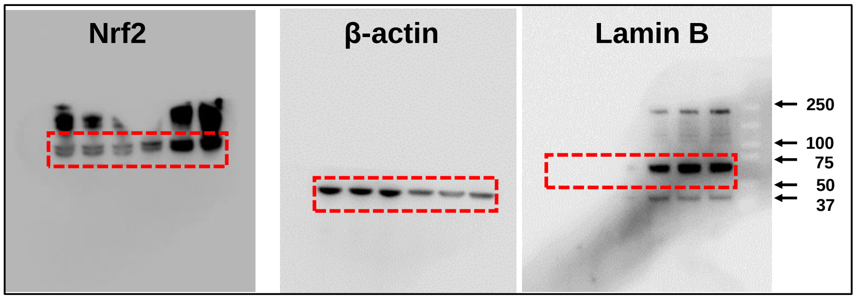


(Continued..)

## Figure 3D

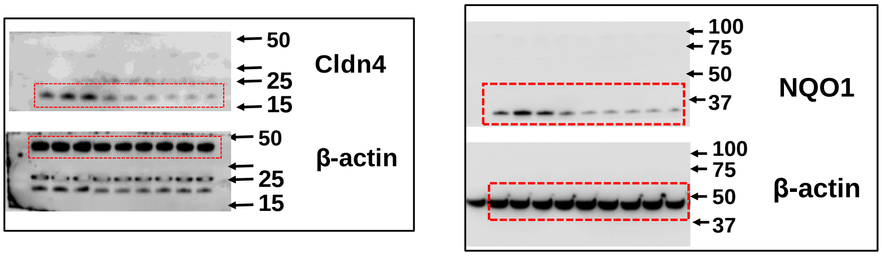


## Figure 3F

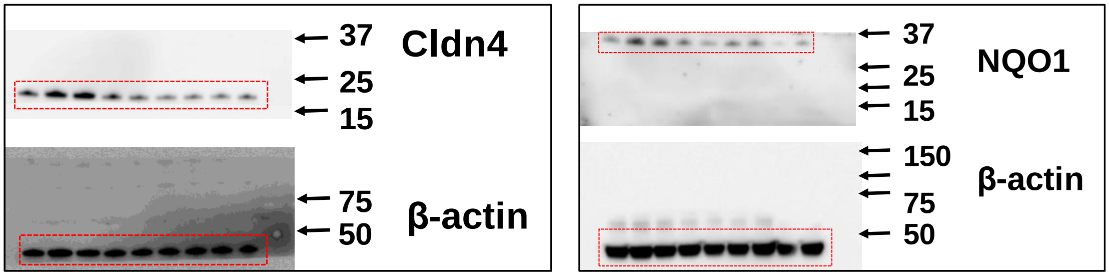


## Figure 4J

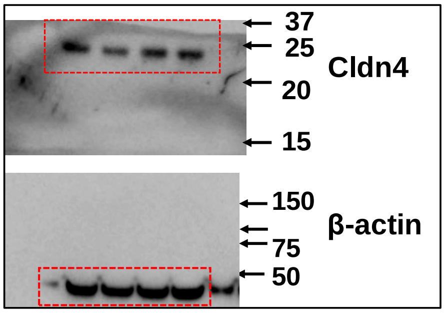


## Supplementary Figure 6A

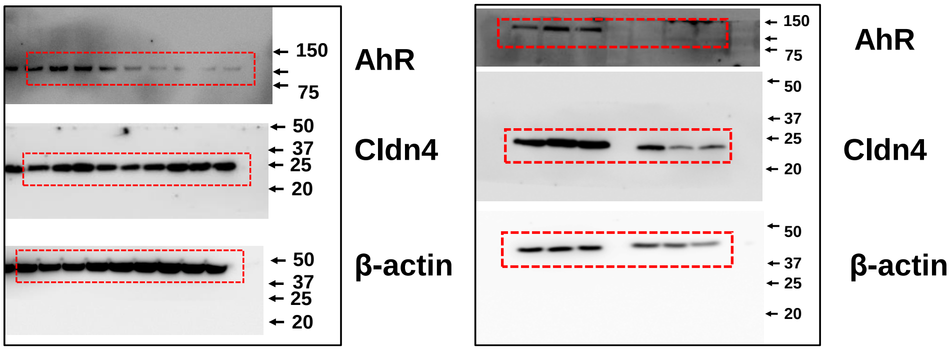


(Continued..)

## Supplementary Figure 6B

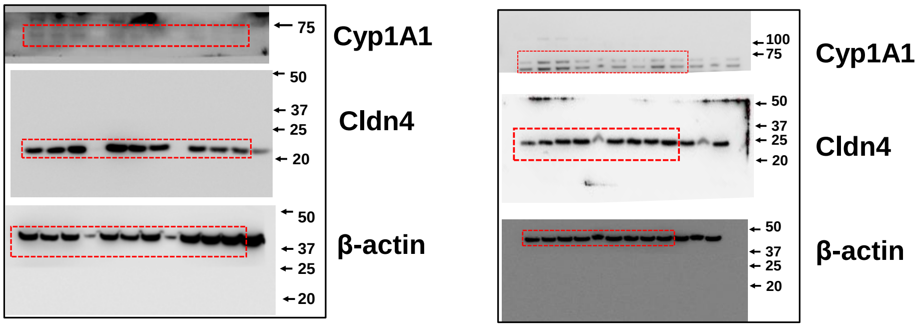


## Supplementary Figure 7A

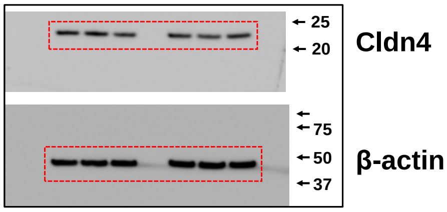


## Supplementary Figure 7C

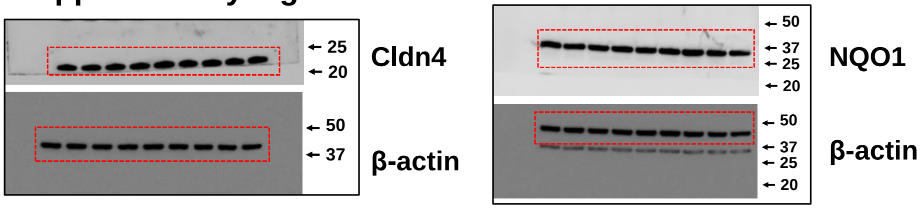


## Supplementary Figure 9D

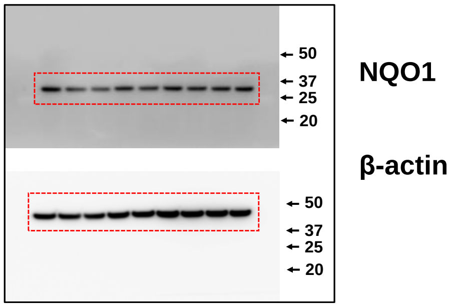


(Continued ..)

## Supplementary Figure 10B

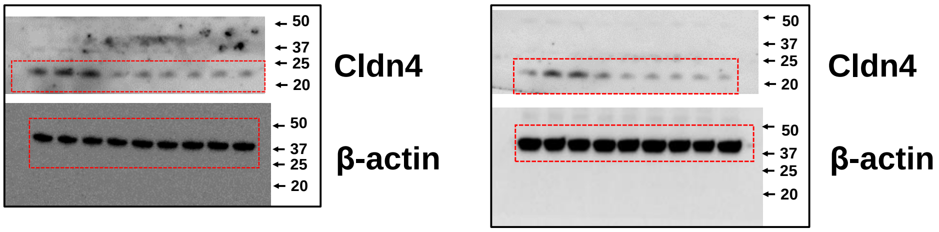


## Supplementary Figure 10C

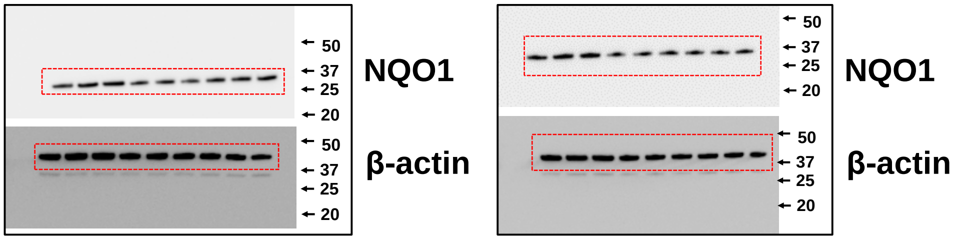


## Supplementary Figure 11A

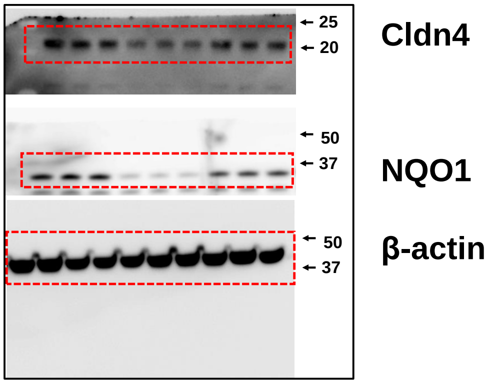


## Supplementary Figure 11B

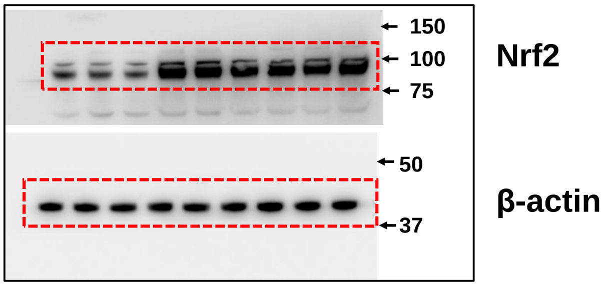


## Supplementary Figure 11D

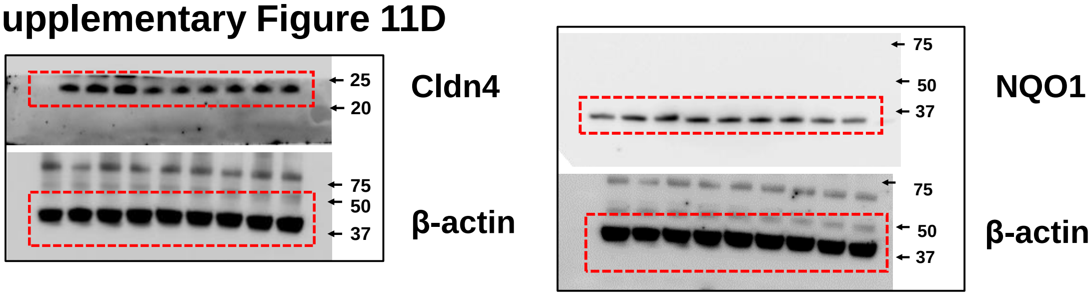

**Supplementary Table 1.** Total raw sequences that are aligned for each sample.

| File | Input Reads | Aligned Reads | Alignment Rate |
|---|---|---|---|
| JV_01_Control_1-40344698 | 29,266,578 | 28,326,908 | 96.8 |
| JV_02_Control_2-40357553 | 29,660,569 | 28,731,371 | 96.9 |
| JV_03_Control_3-40357554 | 20,709,483 | 19,818,098 | 95.7 |
| JV_07_UroA50_1-40357556 | 26,728,324 | 25,856,296 | 96.7 |
| JV_08_UroA50_2-40343749 | 21,782,505 | 20,974,957 | 96.3 |
| JV_09_UroA50_3-40341747 | 27,949,497 | 27,036,215 | 96.7 |
