# Supplementary material for: Enhancement of the gut barrier integrity by a microbial metabolite through the Nrf2 pathway
Source: Nat Commun. 2019 Jan 9;10:89. doi: 10.1038/s41467-018-07859-7 (PMC6327034; doi:10.1038/s41467-018-07859-7)
Supplement: Supplementary file 2 — Description of Additional Supplementary Files [file 41467_2018_7859_MOESM2_ESM.docx]

**Description of Additional Supplementary Files**

**File Name**: Supplementary Data 1

**Description**: Restricted gene list with a q-value of ≤ 0.05. Top 20 down and up regulated gene list is provided in separate sheets. Experimental details are provided in methods.
